# Accurate signal-source localization in brain slices by means of high-density microelectrode arrays

**DOI:** 10.1038/s41598-018-36895-y

**Published:** 2019-01-28

**Authors:** Marie Engelene J. Obien, Andreas Hierlemann, Urs Frey

**Affiliations:** 1 Department of Biosystems Science and Engineering, ETH Zurich, Basel, Switzerland; 2 RIKEN Quantitative Biology Center, Kobe, Japan; 3 MaxWell Biosystems AG, Basel, Switzerland

## Abstract

Extracellular recordings by means of high-density microelectrode arrays (HD-MEAs) have become a powerful tool to resolve subcellular details of single neurons in active networks grown from dissociated cells. To extend the application of this technology to slice preparations, we developed models describing how extracellular signals, produced by neuronal cells in slices, are detected by microelectrode arrays. The models help to analyze and understand the electrical-potential landscape in an *in vitro* HD-MEA-recording scenario based on point-current sources. We employed two modeling schemes, (i) a simple analytical approach, based on the method of images (MoI), and (ii) an approach, based on finite-element methods (FEM). We compared and validated the models with large-scale, high-spatiotemporal-resolution recordings of slice preparations by means of HD-MEAs. We then developed a model-based localization algorithm and compared the performance of MoI and FEM models. Both models provided accurate localization results and a comparable and negligible systematic error, when the point source was in saline, a condition similar to cell-culture experiments. Moreover, the relative random error in the x-y-z-localization amounted only up to 4.3% for z-distances up to 200 μm from the HD-MEA surface. In tissue, the systematic errors of both, MoI and FEM models were significantly higher, and a pre-calibration was required. Nevertheless, the FEM values proved to be closer to the tissue experimental results, yielding 5.2 μm systematic mean error, compared to 22.0 μm obtained with MoI. These results suggest that the medium volume or “saline height”, the brain slice thickness and anisotropy, and the location of the reference electrode, which were included in the FEM model, considerably affect the extracellular signal and localization performance, when the signal source is at larger distance to the array. After pre-calibration, the relative random error of the z-localization in tissue was only 3% for z-distances up to 200 μm. We then applied the model and related detailed understanding of extracellular recordings to achieve an electrically-guided navigation of a stimulating micropipette, solely based on the measured HD-MEA signals, and managed to target spontaneously active neurons in an acute brain slice for electroporation.

## Introduction

Extracellular recording by means of *in vitro* high-density microelectrode arrays (HD-MEAs)^1–9^ has become a powerful tool to resolve subcellular details of single neurons in active networks, grown from dissociated cells, using the spatial information of extracellular action potentials (EAPs). Culturing cells directly on the electrode array allows for detection of neuronal signals along a 2-dimensional plane at high signal-to-noise ratio, so that small axonal signals propagating along axonal arbors can be tracked^10–13^. In brain-slice microelectrode-array (MEA) experiments, however, the active neurons of interest are inside the tissue slab and feature a 3D arrangement, while the viable cells are a few tens of micrometers away from the electrodes^14,15^. Localization of extracellularly recorded active units or neurons in brain tissue is a known challenge, both experimentally and computationally^16–18^. An accurate localization of the detected cells in brain slices could facilitate the classification of the respective cell types and allow for manipulation of target cells by e.g., electroporation, electrical stimulation, local pharmacology application, or other interventions. In this work, we study how signal sources in brain slices can be detected by HD-MEAs in order to establish an accurate localization method of the signal sources. We used a known and simple source in all experiments and models—a point-current source.

To understand the *correlation* between brain slice signals and HD-MEA data, we developed models of the HD-MEA recording environment and, importantly, we compared the models with actual high-spatial-resolution experimental data obtained from HD-MEAs. Previous work by Ness *et al*. presented a modeling-only study, which included slices on traditional MEAs^19^. Assuming an insulating MEA surface, the method of images (MoI)^20–22^ was applied to the volume-conductor theory^20^ in order to analytically compute the electric potentials on the MEA surface. The MoI model was then compared to finite-element-method (FEM) simulations. We here extended the proposed MoI and FEM models by considering factors that we could control in our experimental setup, such as the height of the saline bath, the conductivity of the saline, the location of acute cerebellar slice layers with respect to the point source, and the location of the reference electrode. To compare and validate the models, we performed experiments with both, saline only and acute brain slices in culture solution by using HD-MEAs with 11,011 electrodes (3,150 electrodes per mm^2^)^1^ for recording and a stimulating glass micropipette as a point-current source.

Several factors affect the electrical potential landscape on MEAs in general, whether the signal is coming from a simple point source or from a complex source, such as a neuron. For cell-culture experiments, the amplitude of neuronal signals was observed to increase when mineral oil—a non-conductive fluid normally used to prevent evaporation of culture medium—was poured onto the MEA dish (unpublished data). Similarly, it has been shown that a sheet of glia can serve as an insulator layer and increase the spike amplitude of cultured cells^23^. Microtunnels made of poly(dimethylsiloxane) or PDMS also amplify action potentials propagating along axons^24–26^. In this study, we analyzed how different saline heights, with the saline-to-air interface acting as an insulator, affect the amplitude of MEA signals. For acute brain slice experiments, the reduced conductivity of brain tissue with respect to saline has to be considered and substantially affects the amplitude of the recorded signals. Brain tissue has been mostly considered and modeled as homogeneous and resistive material^21,27,28^.

A thorough understanding of the HD-MEA extracellular environment is a requirement for accurate localization of signal sources in brain slices. In detail, we studied if a realistic HD-MEA model could improve the accuracy (see definition in Methods) of localizing the micropipette in saline and in an acute brain slice. We separated the localization problem into two parts: (a) x-y localization and (b) z localization. We used 2D Gaussian fitting for the x-y localization, assuming the brain slice being homogeneous in the x-y direction, and MoI and FEM models to assess the z-distance (distance between the source and the HD-MEA surface) of the point source in saline and in the presence of an acute brain slice. We fitted the measured potential distribution to the simulated potential distributions of both MoI and FEM models to estimate the z-distances.

To demonstrate an application and the performance of the localization algorithm, we targeted single spontaneously active neurons in acute cerebellar slices for electroporation with a micropipette using a micromanipulator. This experiment served as a proof of concept for solely using the signals of HD-MEAs to steer micropipettes with high accuracy and to demonstrate that an all-electrical electrophysiology and micropipette control system could be realized. Instead of using a microscope to visualize the cells and the micropipette tip, the HD-MEA acted as an electrical-imaging camera to localize both, the electrically active cells and the micropipette. The combined micropipette-HD-MEA approach offers prospects of an electrically guided automated patch-clamp^29^, in contrast to blind^30–32^ and visually^33^ guided approaches. Future applications of this method aim towards high-throughput single-cell manipulation and analysis.

## Results

We developed a localization method that allows for accurate identification of the 3D position of a micropipette, representing a point source, above the HD-MEA surface in the presence of saline or acute brain slices. For simplicity, we separated the localization problem into two parts: x-y localization and z localization. In this way, we were able to extract the x-y location of the point source independently of its z location. Estimating the x-y location is straightforward, based on the peak potential, and we will show that accurate localization can be achieved by Gaussian fitting. For z localization, we performed the following steps: First, we acquired a large experimental HD-MEA data set by recording the signal characteristics and potential landscape of a stimulating micropipette located at various defined z-distances in saline above the microelectrode array. The obtained data were then used to derive an experimentally determined *point spread function* (PSF), and served as a basis for finding the best HD-MEA model for use with the localization algorithm. Second, we modeled the recorded HD-MEA signals by using two methods^19^, (1) analytical equations based on volume-conductor theory and the method of images (MoI), and (2) a finite-element method (FEM). Using these two methods, we explored different parameters that can affect the HD-MEA signals. Third, we determined parameters in the MoI and FEM models that best described our experimental conditions, such as saline column height, tissue thickness and ground location. We then generated data sets for each model by simulating the electrical potential landscape of a stimulating micropipette at z-distances between 1 and 1000 μm at 1 μm increments. Finally, we acquired HD-MEA recording data, while the position of the stimulating micropipette was tracked by the micromanipulator software and confirmed by using microscopy whenever possible (i.e., when the micropipette tip was not inside the slice tissue). We used all these recordings to determine the accuracy of the developed localization method. In a last step, we applied the localization method to target and electroporate individual, spontaneously active neurons in acute slice preparations.

### In-plane localization of micropipettes

To utilize the HD-MEA for locating the position of a micropipette, this micropipette needs to produce an electrical signal, which can be detected by the electrode array. Upon injecting a periodic voltage or current signal (sine or square wave), the micropipette generates an electric field in the conductive medium (saline or culture medium), the potential distribution of which can be measured by the 2D planar electrode array. The signal amplitude on each electrode can be extracted by using fast Fourier transformation (FFT) or demodulation. We applied either sinusoidal or square-wave current signals at 1 kHz frequency, where the tissue shows predominantly Ohmic behavior^34^. We chose this frequency, as it is close to the action-potential (AP) frequency, and as the signal-to-noise ratio (SNR) performance of the HD-MEA is optimized for the AP range^1^. We considered the micropipette tip as a point source^35^ and then *imaged* the electric potential landscape induced by the micropipette.

**Figure 1:**
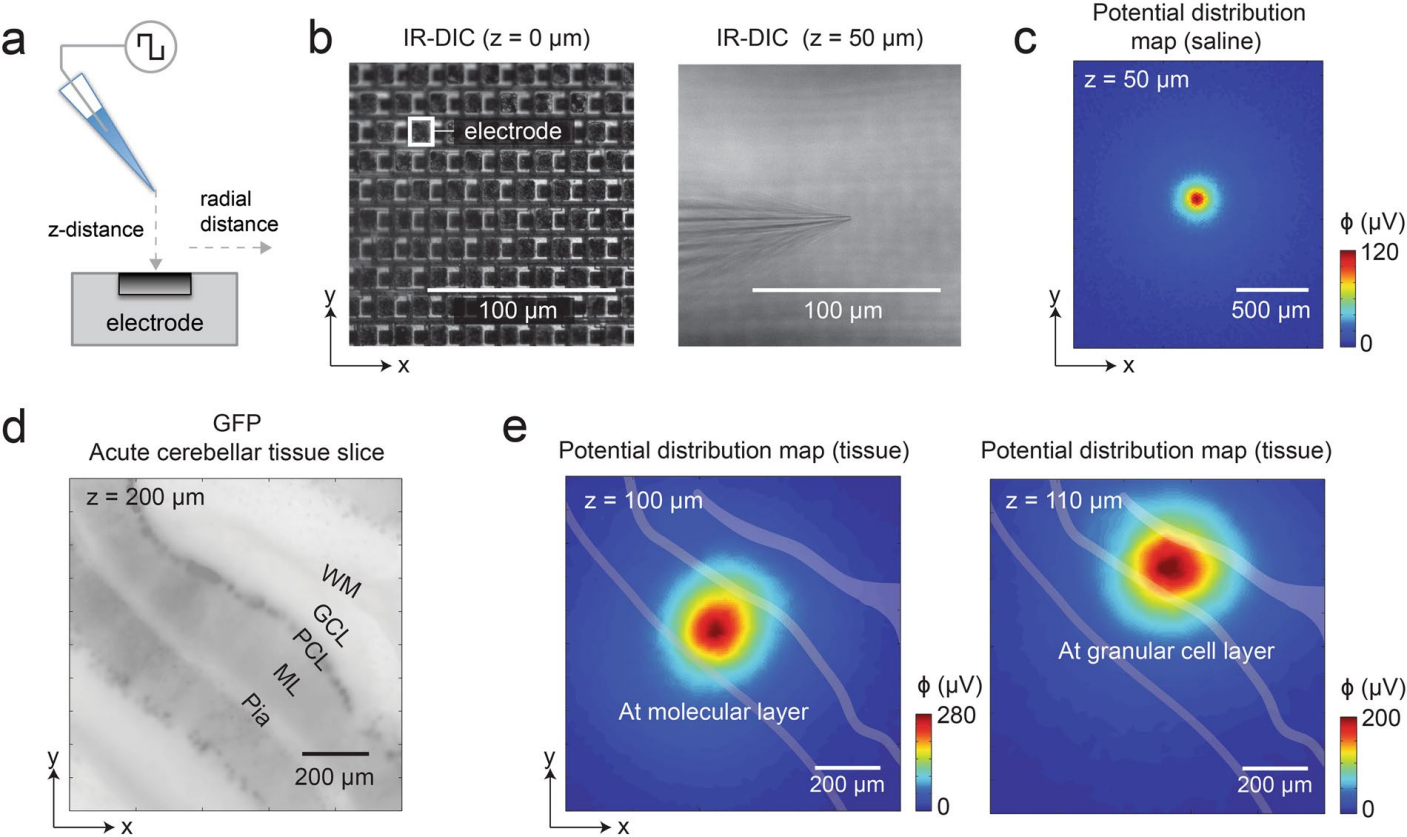
The x-y location of a micropipette can be electrically imaged using an HD-MEA. (**a**) A micropipette tip, stimulating with a current square wave (50 nA, 1 kHz), was used as a point-current source. The electrical potential landscape of the point source was measured in (**b**,**c**) saline and (**d**,**e**) in the presence of an acute brain slice of 200 μm thickness by using an HD-MEA. (**b**) Microscopy images, using infrared differential interference contrast (IR-DIC), show the electrode array (*left panel*) and the micropipette tip (*right panel*). One electrode is highlighted; the electrode size is 10.2 μm × 8.6 μm, the electrodes are arranged in a hexagonal pattern, and the center-to-center distance between the electrodes is 17.8 μm. The HD-MEA surface and the micropipette are at z = 0 and 50 μm, respectively. (**c**) The map illustrates the measured potential spatial distribution of a stimulating micropipette, located at the center of the array at 50 μm z-distance in saline as isotropic conductor medium. The voltage amplitude, detected at each electrode site, is color-coded (blue (0 μV) to red (120 μV)). (**d**) A 200-μm thick-cerebellar slice atop the HD-MEA is imaged by fluorescence microscopy, using a green-fluorescent-protein (GFP) filter. The acute brain slice was obtained from a GAD67-GFP knock-in mouse, where the majority of the GABAergic neurons were GFP positive^36^. The cell bodies can be identified as dark spots in the Purkinje cell layer (PCL), and the dendritic arbors, shown in dark gray, indicate the molecular layer (ML). The other layers were determined according to the established cerebellar structure in literature: pia^39^, granular cell layer (GCL), and white matter (WM). (**e**) The potential spatial distribution maps similar to those in c were obtained by placing a stimulating micropipette at the ML (*left panel*) and at the GCL (*right panel*) of the acute cerebellar slice shown in **(d)**.

The conductor volume above the array surface and its conduction properties shape the electrical field. A homogeneous isotropic medium (e.g., saline or culture medium) will yield a potential that decreases uniformly in all directions (i.e., a symmetric circular potential spread across the MEA), while an inhomogeneous anisotropic medium (e.g., a brain slice) will distort the symmetry of the electric field and result in more complex potential patterns. Figure 1c,e shows amplitude maps measured under different conditions. A sketch of the micropipette above an HD-MEA electrode is shown in Fig. 1a. An upright microscope was used to confirm the location of the micropipette with respect to the HD-MEA surface in saline (Fig. 1b) and in the presence of an acute cerebellar slice (Fig. 1d). Acute brain slices of 200 μm thickness were obtained from a 5-week old GAD67-GFP knock-in mouse, in which a large fraction of GABAergic neurons, such as cerebellar Purkinje cells, were GFP positive^36^. The use of this specific mouse type allowed for identification of the different cerebellar layers in the slice. Amplitude maps, obtained by recording from the full microelectrode array, are shown in Fig. 1c for a micropipette in saline and Fig. 1e for a micropipette in an acute brain slice at two different layers. The square-wave stimulation signal was 50 nA at 1 kHz in all instances. The signal patterns in Fig. 1e were recorded by the electrode array, while the micropipette was in the molecular layer (ML) and granular cell layer (GCL), and evidenced different tissue anisotropies of the cerebellar layers. The location of the peak potential reflects the x-y position of the micropipette tip.

**Figure 2:**
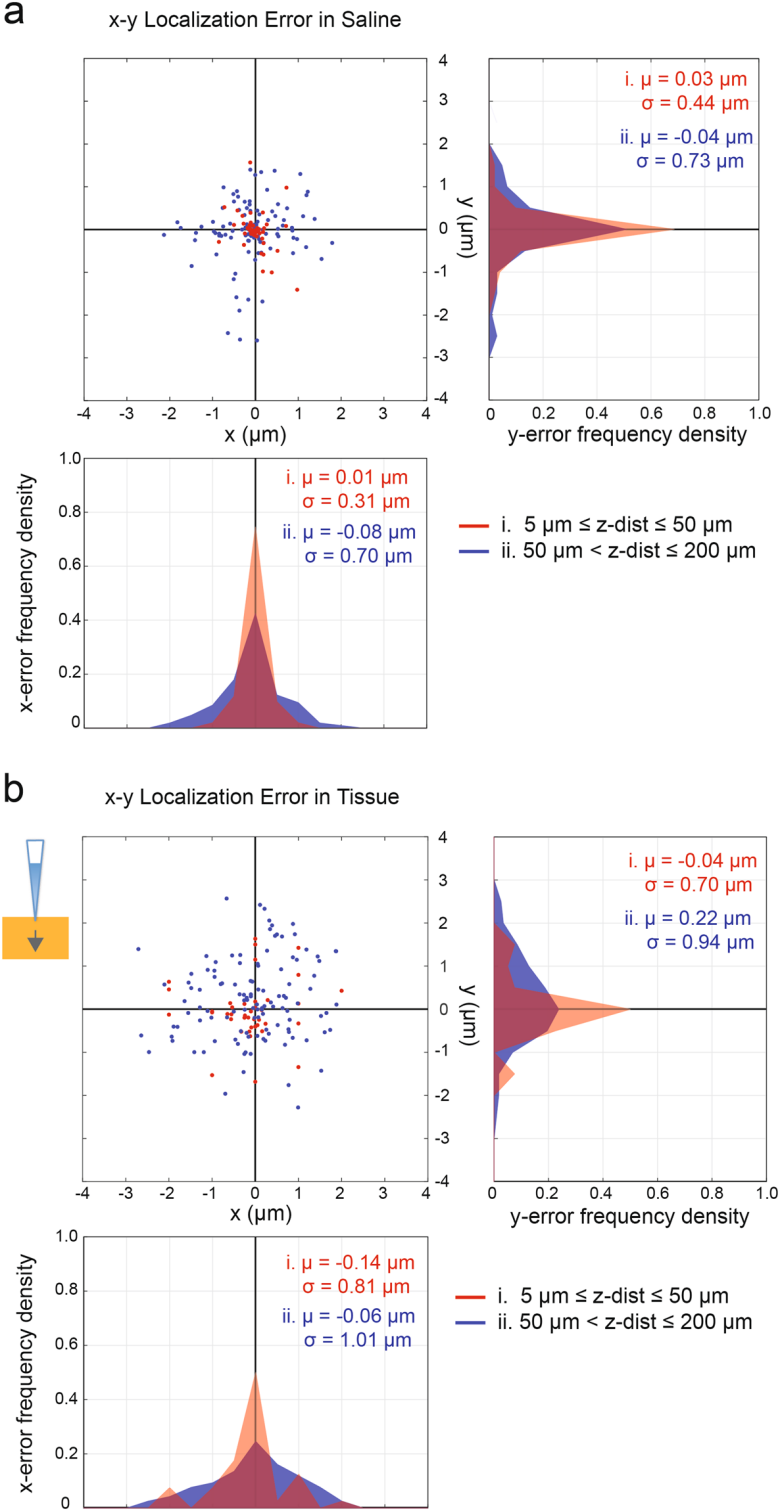
HD-MEA electrical imaging enables x-y localization of a point source in saline and in acute brain slices. The x-y errors of localization experiments for a point source in saline are shown in (**a**). The top left panel shows the x-y errors, when the pipette z distance was (i) 5 μm ≤ z-distance ≤ 50 μm (red dots, N = 51) and (ii) 50 μm < z-distance ≤ 200 μm (blue dots, N = 106). Each dot refers to the difference between measured against estimated x-y location of the pipette. The frequency density histograms of the x-errors (bottom panel) and y-errors (right panel) are shown. The histograms display the number of times a localization estimate yields a certain error value within a bin-width of 0.5 μm for a range of −4 to 4 μm. This error value was normalized with respect to the total number of trials in a specified experimental condition (e.g., saline (i) has 51 trials). (**b**) x-y error results as in a, but obtained from experiments with acute brain slices, where (i) N = 40 and (ii) N = 118. The arrow in the small schematics indicates the pipette movement into the tissue. The x-y errors in b are based on measured x-y locations determined by the manipulator movement and by using calibration data.

We localized the x-y position of the micropipette using two-dimensional Gaussian fitting. Since the search was only for the location of the peak amplitude, a 2D Gaussian least-squares fit was considered sufficient, despite the residuals in the fitting. The results are shown in Fig. 2a. The measured x-y location of the micropipette for each data point corresponds to the programmed micromanipulator position and was confirmed by visual inspection. The target positions were chosen randomly above the electrode array such that the positions were not directly on top of an electrode. We used square-wave (50 nA, 1 kHz) stimulation and recorded for 10 s per location. The data were then analyzed offline to estimate the x-y location from the electrical recordings. We computed the x-y localization errors by subtracting the measured x-y coordinates (microscope and/or micromanipulator data) of the micropipette from the x-y location estimated from the electrode data. The mean and standard deviation (s.d.) of the errors are as follows. When the pipette z-distance was between 5 and 50 μm (N = 51), the mean ± s.d. values were 0.01 ± 0.31 μm and 0.03 ± 0.44 μm, for x and y respectively. The errors increased slightly for pipette z-distances between 50 to 200 μm (N = 106), where the mean ± s.d. were −0.08 ± 0.70 μm for x and −0.04 ± 0.73 μm for y. As expected, the systematic error for these measurements was negligibly small for the full range of z-distances considered, i.e., the mean errors were close to zero. Overall, a single x-y location estimate in saline can be expected to be precise within ±1.46 μm, with 95% confidence.

For experiments with an acute brain slice, we localized the micropipette within the tissue (≤200 μm). In order for the micropipette to move smoothly through the tissue, the micromanipulator was adjusted to approach the HD-MEA surface perpendicularly, i.e., at a 90° angle. The x-y positions were determined only according to micromanipulator movements after calibration. The x-y error results are shown in Fig. 2b, where the mean values ± s.d. amounted to −0.14 ± 0.81 μm and −0.04 ± 0.70 μm for x and y, respectively. On the other hand, for z-distances between 50 to 200 μm, the errors were −0.06 ± 1.01 μm for x and 0.22 ± 0.94 μm for y. Similar to the saline-only case, the systematic error for these measurements in tissue was small (<0.25 μm). Moreover, the precision of x-y localization in tissue is ±2.02 μm, with 95% confidence. To simplify the x-y localization algorithm in brain slices, we assumed homogeneous conductor properties of the tissue in the x-y directions and used 2D Gaussian fitting. The inhomogeneity of the tissue does not create abrupt changes in the potential distribution according to the 2D features of the potential distribution that were observed in cerebellar slice experiments (see also Fig. 1e). The x-y localization errors were low considering the size of the electrodes (10.2 × 8.6 μm^2^) and the distance between electrodes of 17.8 μm. If the x-y localization method will be applied to target single cells, the localization errors are small with respect to the diameter of cell somas (approximately 10 to 30 μm).

### Localization of micropipettes along the z-axis

**Figure 3:**
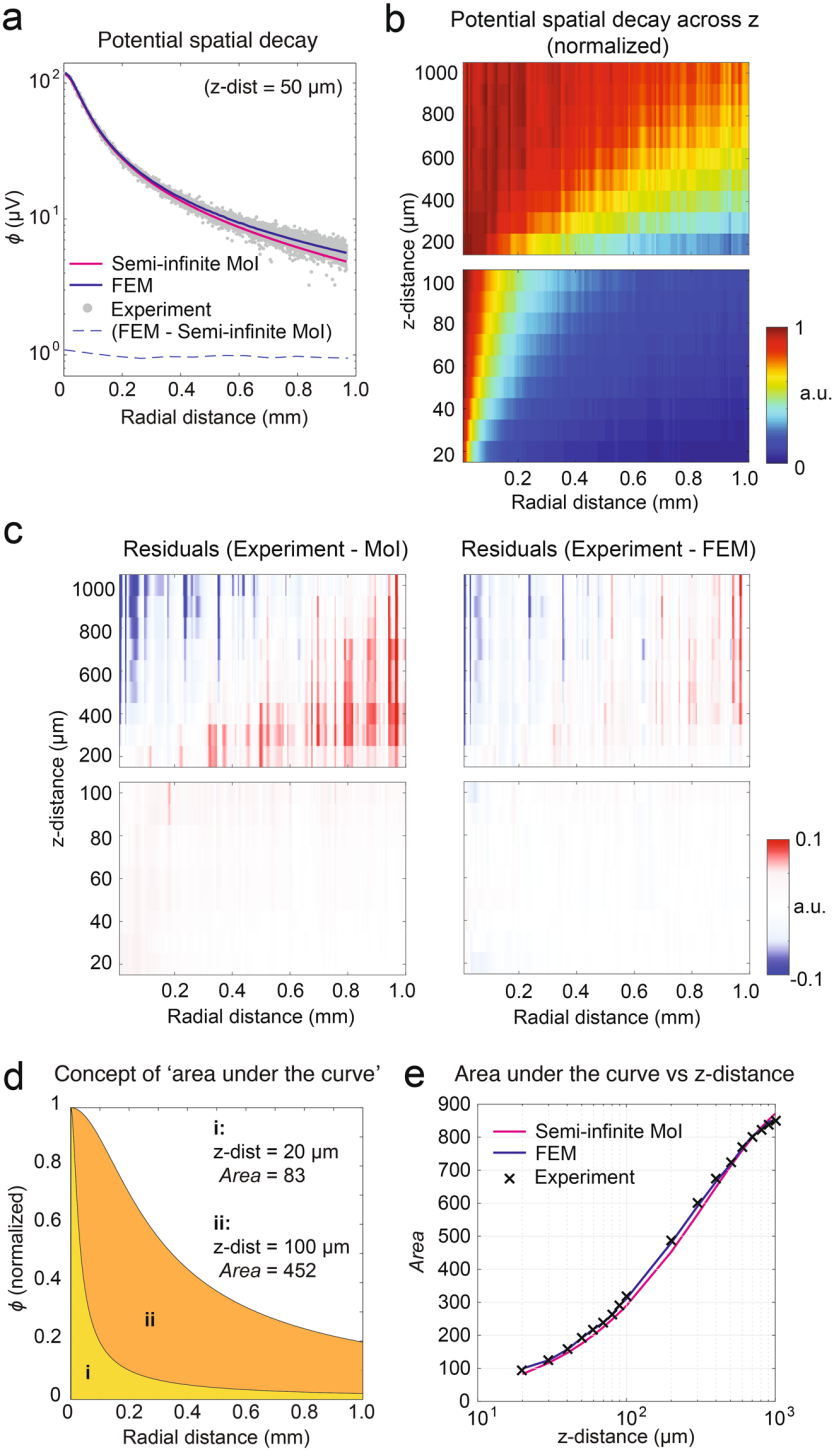
The z-distance between the micropipette and the HD-MEA can be estimated according to the electrical potential spatial distribution. (**a**) Exemplary *potential spatial decay* for a z-distance of the point source of 50 μm including experimental data (gray dots), and data derived from a method-of-images or MoI model (pink line) and a finite-element or FEM model (blue line). The differences between the FEM and MoI values are displayed as a blue dashed line. The gray dots represent potential values measured through the 11’000 electrodes shown in Fig. 1c. (**b**) Experimentally determined potential spatial distributions, normalized to the maximum potential value per z-distance. (**c**) Residuals computed by subtracting the normalized potential spatial decays of experiments and models: MoI (left) and FEM (right). In general, the FEM model residuals are closer to zero. (**d**,**e**) Comparison of potential spatial decays according to the area under the curve (*Area*), computed by numerical integration over the interval of 1 to 1000 μm distance from the peak value. The *Area* provides information on the steepness of the curve: the slope is infinite if the *Area* is zero and the slope is zero if the *Area* is 1000. (**d**) Two MoI-based potential spatial decays (normalized) for z-distances of 20 μm (i) and 100 μm (ii), are plotted and the computed *Area values* amounted to 83 and 452, respectively. (**e**) Values of *Area* computed for z-distances between 20 and 1000 μm, based on normalized potential spatial decays obtained from experiments, MoI, and FEM. *Area* values derived from the FEM model were closer to the experimental results.

Aside from estimating the x-y location of the micropipette on the array, the electrical potential distribution reflects the z-distance of the signal source. In order to obtain a point spread function (PSF) on the HD-MEA, we measured the electrical potential distribution for z-distances ranging from 20 to 1000 μm. Figure 3a (z-distance of 50 μm) and **b** (all z-distances) show the electrical-potential-spatial-decay curves obtained from the experiments. An electrical *potential spatial decay* comprises of all extracted electrical potential values, plotted as a function of the radial distance of the recording electrode (along the x-y plane) to the location of the maximum amplitude. The potential spatial decays were normalized to the maximum potential for each z-distance. We compared the potential spatial decays obtained from the experiments to the modeling results and show the residuals in Fig. 3c (see next subsections for the description of the models).

We quantified the steepness of the potential spatial decay curves by computing the area under the curve (denoted as “Area”). The Area under each curve was obtained by numerical integration of the amplitude values in an interval of 1 to 1000 μm distance from the peak amplitude. When the slope of the curve is infinite, the Area is one; on the other hand, if the Area value is 1000, the curve is flat (zero slope). This approach allows for generalizing the potential spread in dependence of the z-distance, irrespectively of the magnitude of the signal amplitude. Figure 3d,e present the Area results under saline conditions.

#### HD-MEA models: method of images

The method of images (MoI) provides an analytical solution to the potential in a volume conductor involving boundary surfaces, which apply to HD-MEA measurements^19–22,35^. We will introduce details of the MoI concept in the Methods section and we summarized the equations used in this work in Tables 1 and 2. In brief, MoI replaces the boundaries with virtual point current sources located at appropriate positions to meet the respective boundary conditions. We refer the readers to literature^19,37^ for a more detailed discussion and derivations of the equations. We first considered the following cases. The HD-MEA surface was modeled as an electrical insulator with the following boundary conditions: (i) a semi-infinite conductor model in the z-direction considering the insulating electrode array surface (Equation 2) and (ii) a bounded model with two boundaries, i.e., the insulating array surface and the liquid-to-air interface (Equation 3). The potential *ϕ*_*se*_, induced by a point current source *I* located at (*x*, *y*, *z*), can be calculated at any point (*x*′, *y*′, 0) on the electrode array using the equations in Table 1. The conductivity *σ*_*s*_ refers to saline medium. For all simulations based on the described models, we used the same current amplitude *I* as in the experiments, 50 nA. The conductivity values used in all MoI models are given in Table 3.

**Table 1:** Equations to model HD-MEA signals in isotropic media.

Model	Equation	Assumption	Use
Volume conductor theory	**Equation 1** ${{\rm{\varphi }}}_{{{\rm{\sigma }}}_{{\rm{s}}}}({\rm{x}}^{\prime} ,{\rm{y}}^{\prime} ,{\rm{z}}^{\prime} )=\frac{{\rm{I}}}{4{\rm{\pi }}{\rm{\sigma }}\sqrt{{({\rm{x}}^{\prime} -{\rm{x}})}^{2}+{({\rm{y}}^{\prime} ,-{\rm{y}})}^{2}+{({\rm{z}}^{\prime} -{\rm{z}})}^{2}}}$ where *σ* = *σ*_*s*_.	No boundaries	First-order approximation of extracellular recordings without the MEA surface
Semi-infinite MoI	**Equation 2** ${{\rm{\varphi }}}_{{\rm{se}},{{\rm{\sigma }}}_{{\rm{s}}}}({\rm{x}}^{\prime} ,{\rm{y}}^{\prime} ,0)=2\ast {\varphi }_{{{\rm{\sigma }}}_{{\rm{s}}}}({\rm{x}}^{\prime} ,{\rm{y}}^{\prime} ,0)$	Single boundary (HD-MEA surface as insulator)	First-order approximation of *in vitro* MEA signals
Bounded MoI, (z < *h*_*s*_)	**Equation 3** ${{\rm{\varphi }}}_{{\rm{bo}},{\rm{z}} < {{\rm{h}}}_{{\rm{s}}}}({\rm{x}}^{\prime} ,{\rm{y}}^{\prime} ,0)={\varphi }_{{\rm{se}},{{\rm{\sigma }}}_{{\rm{s}}}}({\rm{x}}^{\prime} ,{\rm{y}}^{\prime} ,0)+{{\rm{\gamma }}}_{{\rm{s}}}$ where ${{\rm{\gamma }}}_{{\rm{s}}}=2\sum _{{\rm{n}}=1}^{\infty }\,{{\rm{W}}}_{{\rm{SA}}}^{{\rm{n}}}[{\varphi }_{{{\rm{\sigma }}}_{{\rm{s}}}}({\rm{x}}^{\prime} ,{\rm{y}}^{\prime} ,-\,2{{\rm{nh}}}_{{\rm{s}}})+{\varphi }_{{{\rm{\sigma }}}_{{\rm{s}}}}({\rm{x}}^{\prime} ,{\rm{y}}^{\prime} ,2{{\rm{nh}}}_{{\rm{s}}})]$, *W*_*SA*_ = 1 and *h*_*s*_ is the height of the saline.	Double boundaries (HD-MEA surface and liquid-air interface as insulators)	*In vitro* MEA model considering liquid column height

**Table 2:** Equations to model HD-MEA signals under tissue slices and with saline cover^19,37,59,72,73^.

Model	Equation
Bounded MoI, isotropic tissue, (z < *h*_*T*_)	**Equation 4** ${{\rm{\varphi }}}_{{\rm{bo}},{\rm{z}} < {{\rm{h}}}_{{\rm{T}}}}({\rm{x}}^{\prime} ,{\rm{y}}^{\prime} ,0)={{\rm{\varphi }}}_{{\rm{se}},{{\rm{\sigma }}}_{{\rm{T}}}}({\rm{x}}^{\prime} ,{\rm{y}}^{\prime} ,0)+{{\rm{\gamma }}}_{{\rm{TS}}}+{\rm{C}}$ where ${{\rm{\gamma }}}_{{\rm{TS}}}=2\sum _{{\rm{n}}=1}^{\infty }\,{{\rm{W}}}_{{\rm{TS}}}^{{\rm{n}}}\,[{{\rm{\varphi }}}_{{{\rm{\sigma }}}_{{\rm{T}}}}({\rm{x}}^{\prime} ,{\rm{y}}^{\prime} ,-\,2{{\rm{nh}}}_{{\rm{T}}})+{{\rm{\varphi }}}_{{{\rm{\sigma }}}_{{\rm{T}}}}({\rm{x}}^{\prime} ,{\rm{y}}^{\prime} ,2{{\rm{nh}}}_{{\rm{T}}})]$, *W*_*TS*_ = (*σ*_*T*_ − *σ*_*S*_)/(*σ*_*T*_ + *σ*_*S*_), *σ*_*T*_ is the conductivity of the acute tissue, *σ*_*S*_ corresponds to the saline cover and *h*_*T*_ is the thickness of the tissue. The conductivity used in ${{\rm{\varphi }}}_{{\rm{se}}}$ is *σ*_*T*_. *C* is the offset due to the height *h*_*S*_ of the saline cover.
Semi-infinite MoI, anisotropic tissue ${{\rm{\sigma }}}_{{{\rm{T}}}_{{\rm{a}}}}$	**Equation 5** ${{\rm{\varphi }}}_{{\rm{se}},{{\rm{\sigma }}}_{{{\rm{T}}}_{{\rm{a}}}}}({\rm{x}}^{\prime} ,{\rm{y}}^{\prime} ,0)=\frac{{\rm{I}}}{2{\rm{\pi }}\sqrt{{{\rm{\sigma }}}_{{\rm{Ty}}}{{\rm{\sigma }}}_{{\rm{Tz}}}{({\rm{x}}^{\prime} -{\rm{x}})}^{2}+{{\rm{\sigma }}}_{{\rm{Tx}}}{{\rm{\sigma }}}_{{\rm{Tz}}}{({\rm{y}}^{\prime} -{\rm{y}})}^{2}+{{\rm{\sigma }}}_{{\rm{Tx}}}{{\rm{\sigma }}}_{{\rm{Ty}}}{({\rm{z}}^{\prime} -{\rm{z}})}^{2}}}$
Bounded MoI, anisotropic tissue ${{\rm{\sigma }}}_{{{\rm{T}}}_{{\rm{a}}}}$, (z < *h*_*T*_)	**Equation 6** ${{\rm{\varphi }}}_{{\rm{bo}},{\rm{z}}^{\prime} < {\rm{h}}}({\rm{x}}^{\prime} ,{\rm{y}}^{\prime} ,0)={{\rm{\varphi }}}_{{\rm{se}},{{\rm{\sigma }}}_{{{\rm{T}}}_{{\rm{a}}}}}({\rm{x}}^{\prime} ,{\rm{y}}^{\prime} ,0)+{{\rm{\gamma }}}_{{{\rm{T}}}_{{\rm{a}}}}+{\rm{C}}$ where ${{\rm{W}}}_{{{\rm{T}}}_{{\rm{a}}}{\rm{S}}}\equiv \frac{\sqrt{{{\rm{\sigma }}}_{{\rm{Tx}}}{{\rm{\sigma }}}_{{\rm{Ty}}}}-\sqrt{{{\rm{\sigma }}}_{{\rm{Sx}}}{{\rm{\sigma }}}_{{\rm{Sy}}}}}{\sqrt{{{\rm{\sigma }}}_{{\rm{Tx}}}{{\rm{\sigma }}}_{{\rm{Ty}}}}+\sqrt{{{\rm{\sigma }}}_{{\rm{Sx}}}{{\rm{\sigma }}}_{{\rm{Sy}}}}}$ assuming that both tissue and saline have similar planar anisotropy, i.e., *σ*_*Tx*_/*σ*_*Ty*_ = *σ*_*Sx*_/*σ*_*Sy*_.

**Table 3:** Conductivity values used in the MoI and FEM HD-MEA models^38,39^.

Volume conductor	Conductivity	Model values (S/m)
Saline (PBS)	*σ*_*S*_	1.30
Saline (ACSF)	*σ*_*S*_	1.18
Tissue (isotropic)	*σ*_*T*_	0.30
Acute cerebellar slice, granular-cell layer (GCL)	*σ*_*Tx*_	0.20
	*σ*_*Ty*_	0.17
	*σ*_*Tz*_	0.18
Molecular layer (ML)	*σ*_*Tx*_	0.20
	*σ*_*Ty*_	0.28
	*σ*_*Tz*_	0.33

Adding an acute brain tissue on top of the HD-MEA introduces inhomogeneity in the volume conductor. A tissue slice features inhomogeneous and anisotropic conductivity. Nevertheless, in a first-order approximation, we modeled the slice as a homogeneous tissue volume and introduced changes in conductivity at the boundaries of the slice. The x-y dimensions of a brain slice are typically much larger than its z-dimension, and measurements are mostly conducted near the center area of the slice, which is sufficiently far away from the tissue volume lateral boundaries. We therefore assumed a slice of infinite extension in x-y dimension and only included interface boundaries in the z direction. Similar to the saline case above, we considered two boundaries: the HD-MEA surface and the tissue-saline interface. The effect of a third interface, which represented the saline-air interface, was assumed to be a constant value *C* in the tissue MoI model. We present the MoI models for a brain tissue conductor in Table 2. The main difference between the bounded MoI models for saline (Equation 3) and for tissue (Equation 4 and Equation 6) included the conductivity changes at the tissue-saline boundary. In Equation 3, the weighting factor, *W*_*SA*_, is a constant value equivalent to 1 for the saline-air interface, while in Equation 4 the factor for a tissue-saline interface has negative values for all odd *n’s* and positive values for all even *n’s*. Therefore, the series rapidly converges for the tissue-saline bounded MoI model, and a finite number of terms for the summation can be chosen^19^. In this work we summed, similar to Ness *et al*., over 20 terms for bounded MoI models with tissue conductor. The effect of the tissue-saline boundary becomes negligible when the z-distance becomes ≪*h*_*T*_.

#### HD-MEA models: finite-element method

For an even more realistic HD-MEA representation, including the effects of grounding and the shape of the chamber hosting the liquid, we created a finite-element-method (FEM) model using COMSOL Multiphysics version 5.2 and COMSOL with Matlab. A descriptive sketch of the HD-MEA FEM model is shown in Supplementary Fig. S03a. The 3D model geometry consisted of the saline bath (Supplementary Fig. S03b) and the acute brain slice (Supplementary Fig. S03c). Two versions of the acute slice model were created, one that was isotropic and another one, which was anisotropic and inhomogeneous. The HD-MEA surface, at which the electrical-potential distribution and voltage signal amplitudes were computed, was placed in the center of the chamber.

We set the electrical conductivity of the saline and tissue domains in the model based on measured conductivity values and literature. We summarized the conductivity values used for MoI and FEM simulations in Table 3. For saline, we used a 4-pole conductivity probe (SevenGo SG23 with InLab 738-ISM, Mettler Toledo, Ohio, US) to measure the conductivity of the solution (phosphate-buffered saline, PBS) to σ_*S*_ = 1.31 ± 0.20 S/m. The entire chamber (4 mm height, 8.5 mm radius) was filled with saline in the FEM model as in the experiments. For the acute-slice model, we also measured the conductivity of the saline covering the slice (artificial cerebrospinal fluid, ACSF), which was σ_*S*_ = 1.18 ± 0.13 S/m. We simplified our cerebellar-slice model by creating two adjoining boxes corresponding to two cerebellar layers, the granular-cell layer (GCL) and molecular layer (ML). The thickness of the boxes was 0.2 mm, which was the same thickness as that of the slices in the experiments. However, the conductivity and anisotropy of the acute cerebellar slices dissected from mice were unknown. For both the MoI and FEM models, we used values based on previous measurements of the tissue conductivity in turtle^38^ and cat^39^ cerebellar cortices. We show in Supplementary Fig. S06 that FEM simulations using the conductivity values in Table 3 come very close to the experimental data.

The stimulating micropipette was considered a point-current source. The micropipette itself was not modeled, as we considered the associated effects to be minimal. In our acute slice experiments, we initially identified the relative location of the point source with respect to the cerebellar layers and the HD-MEA. This allowed us to model the location of the point source in the tissue as accurate as possible.

We defined the liquid-air interface and MEA chamber surface as electric insulators. The inner wall of the chamber was set to ground (boundary condition: *ϕ* = 0), as a platinum ring attached to the chamber wall was used as the reference electrode in the experimental setup.

The MEA surface itself is mostly insulating in nature (Si_3_N_4_), but a significant fraction (28%) consists of highly conductive microelectrodes. Important to note here is, that the electrodes are connected to recording amplifiers with a high input impedance of >40MΩ at 1 kHz, which limits the actual current following through the amplifier. In addition, we used only 126 connected electrodes of the HD-MEA at any time, leaving the other electrodes floating. Therefore, the effective conducting area is reduced to 0.3% with a total impedance of above 320 kΩ. In consequence, we considered the electrode array as an insulator in the FEM model.

#### Effect of liquid height on the potential spatial distribution

We used the bounded MoI to investigate the effect of the liquid column height to the potential spatial distribution of a point source on the HD-MEA. We computed the potential spatial decay for different z-distances (from 10 to 1000 μm) and increasing liquid column height (1, 2, 4, 8 mm). The potential offsets in the bounded MoI are a consequence of the second part of *Equation 3*, wherein the denominators are dominated by the liquid column height *h*_*s*_. It is important to note that *Equation 3* involves a summation of a harmonic series, which diverges^40,41^. All MoI equations assume the reference electrode or ground being at infinite distance from the point source and from the electrode locations, where the potential amplitudes were simulated. Analytically, the infinite number of terms, summed in Equation *3*, corresponds to the assumption of a ground electrode at infinite distance, around a disk-shape volume of conducting medium, sandwiched between two insulator surfaces. Practically, however, the HD-MEA chamber’s physical dimensions entail that the ground electrode is within millimeters from the point source. The assumption of an infinitely distant ground electrode does not hold. In order to introduce the grounding effect, the potential at the chamber ring, *ϕ*_ring_, can be subtracted from the potential values across the spatial extension. With this analytical solution for the grounding effect, a high number of terms can be chosen, e.g., 100 terms_._ We tested this procedure for a z-distance of 50 μm and obtained a potential distribution with values that were similar to those obtained with FEM. We applied the grounding effect to all bounded-MoI results.

**Figure 4:**
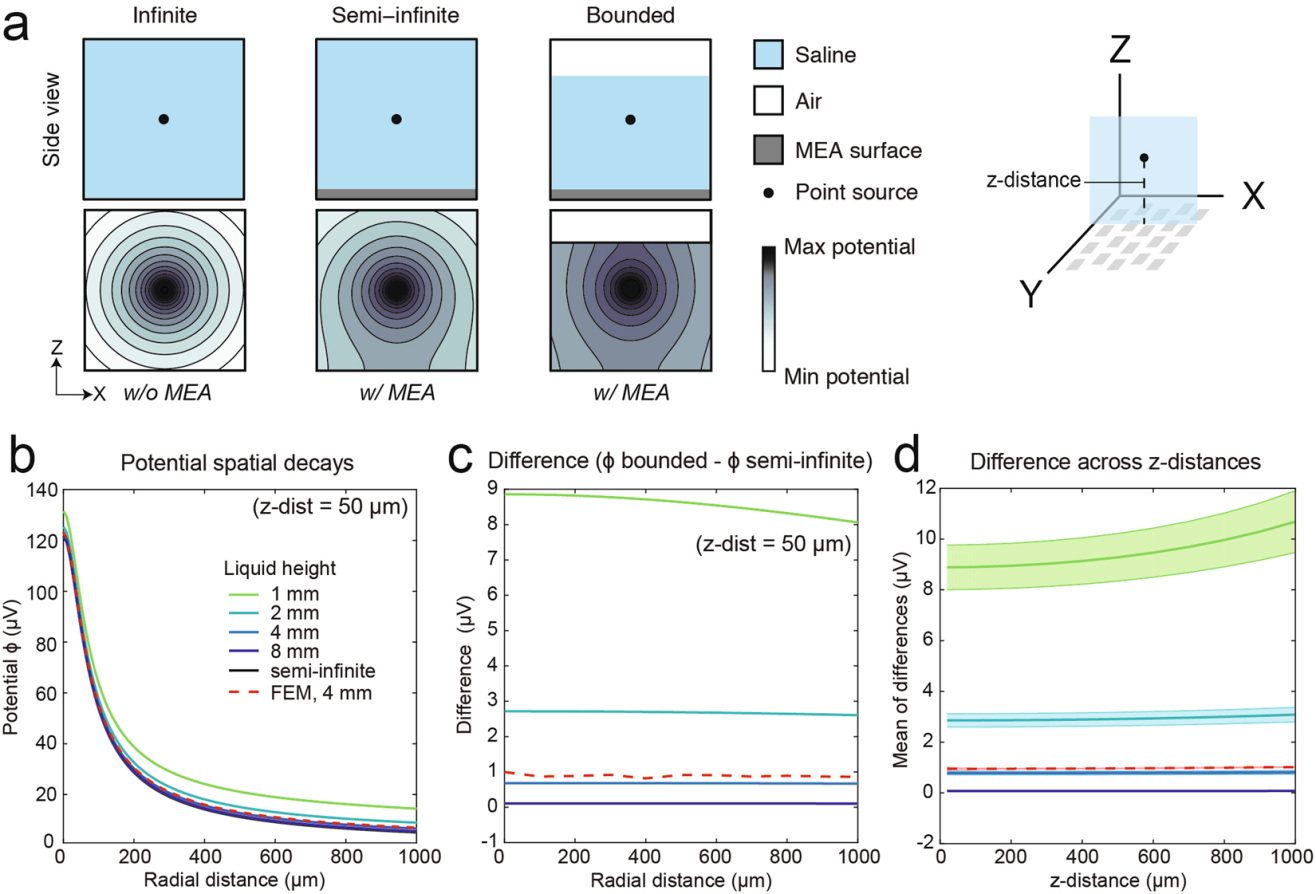
Saline column height introduces an approximately constant increase in potential across the MEA surface. (**a**) Overview of the method-of-images (MoI) models. The top panels show the model boundaries, wherein *infinite* has no boundary, *semi-infinite* has an insulating boundary at the bottom corresponding to the HD-MEA surface, and *bounded* has an additional insulating boundary at the top corresponding to the liquid-air interface. The bottom panels illustrate the radial electric potential distributions. The insulating boundaries massively influence the potential distribution and landscape. The magnitude of the electric potential, detected at the HD-MEA surface, was increased upon adding an insulating boundary (liquid-air-interface). (**b**) Potential spatial decays versus radial distance for a point source at a z-distance of 50 μm, simulated for different liquid column heights (1, 2, 4, 8 mm and semi-infinite). The FEM result is also shown (red dashed line). The magnitude of the electric potential was highest for 1 mm liquid column height. (**c**) Difference in the electric potential spatial decays between bounded MoI (1, 2, 4, 8 mm) and semi-infinite MoI. The difference between FEM and semi-infinite MoI is also included. The potential increase in the bounded case is approximately constant along the radial distance on the HD-MEA surface. As the liquid height decreases, the difference in the potential spatial decays of MoIs (bounded - semi-infinite) increases. (**d**) The mean values and standard deviations of the differences were computed for z-distances between 10 and 1000 μm and are represented as solid lines and shadowing. As the point source gets closer to the upper boundary, i.e., the liquid-air interface, the potential increases as indicated by the results for 1 mm liquid column height. All bounded MoI results include the grounding effect.

We compared the bounded-MoI results with that of a semi-infinite MoI featuring infinite liquid column height. The results are presented in Fig. 4. We first illustrate the electric fields simulated using different analytical models: infinite (no HD-MEA), semi-infinite MoI, and bounded MoI (Fig. 4a). The magnitude of the potential, measured at a certain location on the electrode array surface, increased when an additional boundary was introduced. As expected from Equation 3, the potential values increased upon lowering the liquid height. In Fig. 4b, we present the potential spatial decays simulated for a z-distance of the point source of 50 μm. The increase in potential due to lowering the liquid column height was found to be nearly constant across the lateral distance range on the array surface (Fig. 4c). This means that the potential spatial decay only differed by an offset, while the shape of the curves was very similar. The modeling indicated that the spatial features of the recorded neuronal EAPs by HD-MEAs seem to be largely independent of the liquid column height.

#### Effect of brain tissue to the potential spatial distribution

**Figure 5:**
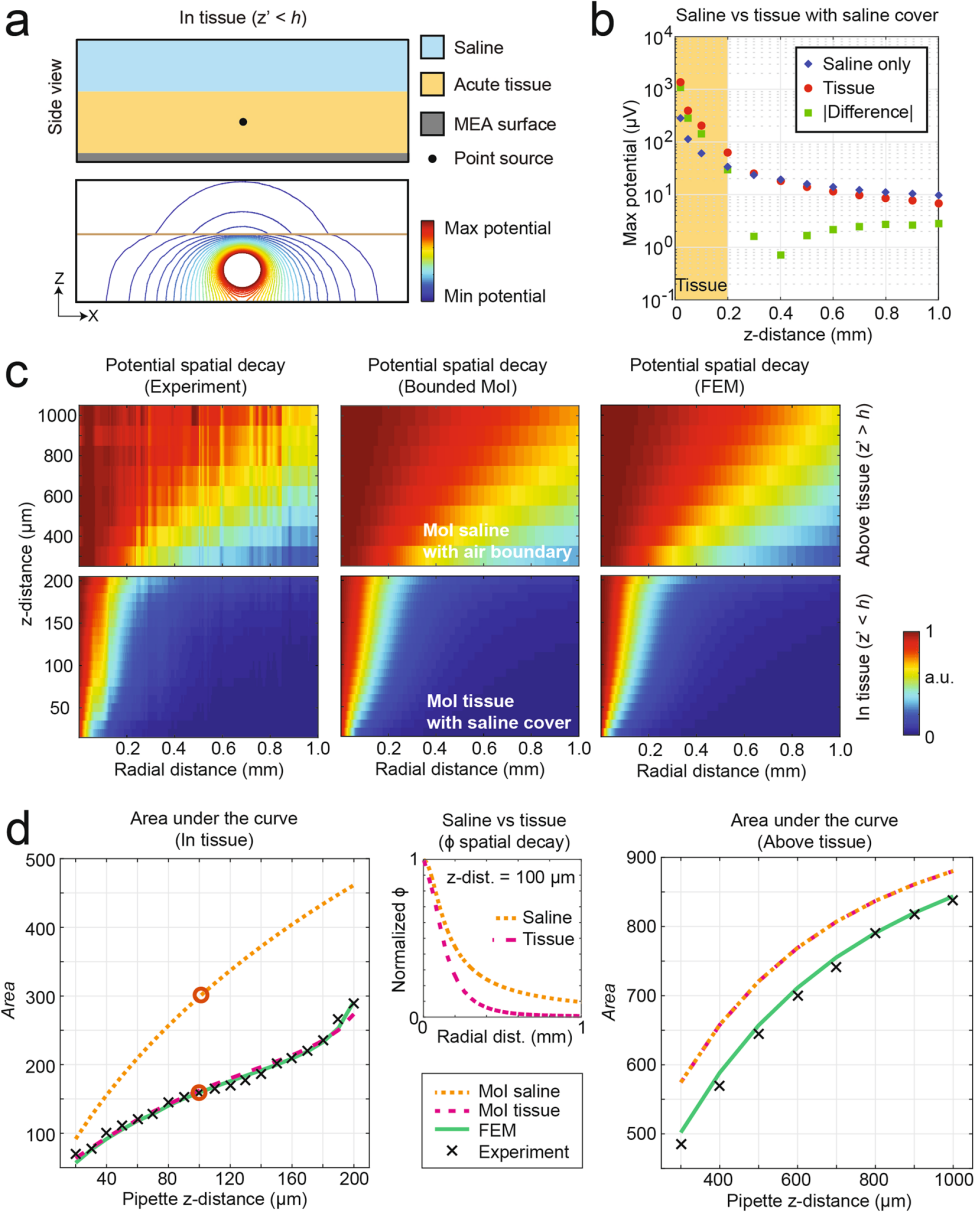
The brain slice has an effect on the potential distribution compared to saline only. (**a**) Illustration of the electric potential distribution if the point current source is in the tissue. The top panel shows the MEA surface as an insulating boundary, the tissue-saline boundary, and the location of the point source for the simulated electric potential distribution that is shown in the bottom panel. (**b**) Comparison of the maximum potential values measured in saline only versus in a tissue slice with saline cover. While the point source is above the tissue, there is no significant difference between the potential values. When the point source is in the tissue, the increased of resistivity of the brain slice causes an increased magnitude of the potential detected at the MEA surface. (**c**) Potential spatial decay plots, showing the relative potential values normalized to the maximum potential per z-distance, obtained from experiments (left), MoI (middle), and FEM (right). (**d**) Area under the curve (*Area*) versus z-distance, computed by numerical integration of the normalized potential over the interval between 1 and 1000 μm lateral distance from the peak value. The figure shows *Area* values, derived from the MoI in saline as in Fig. 3 (orange dotted line), MoI in tissue (pink dashed line), FEM (green line), and experimental data (black x**’**s). The potential spatial decay is steeper for a lower *Area* value, as shown in the example for a z-distance of 100 μm (middle panel). The corresponding *Area* values are highlighted by circles on the left panel.

The placement of an acute brain slice on the HD-MEA introduces a less conductive layer between the HD-MEA surface and the saline. The electrical potential distribution obtained from a point-current source within the tissue is illustrated in Fig. 5a. The tissue exhibits larger resistivity and different dielectric properties as compared to saline. Therefore, the electrical potential distribution, detected at the HD-MEA surface, is different from that in the presence of a highly conductive culture medium or saline. The first brain-tissue effect we observed was the increase in potential measured by the HD-MEA and originating from the stimulating micropipette. We compared the maximum potential, recorded by the HD-MEA, when the micropipette was placed at different z-distances in pure saline and in an acute brain slice with a saline cover (Fig. 5b). As the micropipette reached the tissue (z = 200 μm), the potential, detected by the HD-MEA, increased to approximately twice the potential measured in saline. The increase in magnitude of the measured potential grew exponentially as the micropipette was positioned deeper into the tissue, closer to the electrode array surface.

We further investigated, if the increase in potential due to the brain tissue was constant across the array, or if the steepness of the potential spatial decay was affected. The potential spatial decays based on experiments and extracted from both models, MoI and FEM, are shown in Fig. 5c. We used the bounded MoI model in two versions: (i) with the point source being above the tissue, in which the MoI lower and upper boundaries included the HD-MEA surface and the liquid-air interface, and (ii) with the point source being within the tissue, in which the MoI lower and upper boundaries were the HD-MEA surface and the tissue-saline interface, respectively. Similar to the saline case in Fig. 5, we computed the area under the curve or “Area” for all the potential spatial decays obtained from experiments and models. Interestingly, when the point source was within the tissue, the Area values were lower compared to just saline (Fig. 5d, left panel), indicating a steeper potential decay in the tissue. An example is presented in Fig. 5d (center panel) for z-distance at 100 μm, showing a sharper potential spatial decay when the point source was in tissue as compared to saline. When the point source was above the tissue, the Area values were also lower compared to saline Fig. 5d (right panel). This means that the acute brain slice, which adds an interface boundary in the volume conductor close to the HD-MEA surface, introduces a ‘focusing effect’ on the potential spatial decay.

We illustrate the ‘focusing effect’ of the brain slice in Supplementary Fig. S05. This ‘focusing effect’ can analytically be explained using the concept of MoI. The second interface boundary, i.e. tissue-to-saline interface, leads to additional mirrored point sources above the MEA surface (see *ϒ*_*TS*_ in Equation 4 and Supplementary Fig. S05a). The first set of additional mirrored point sources is within 600 μm from the array surface for a brain slice thickness of 200 μm. For example, the mirrored equivalent point sources due to the tissue-to-saline boundary for a point source, located at z = 50 μm, are located at z-distances of 350 μm (2h_T_ − z) and 450 μm (2h_T_ + z), where h_T_ is the height of the tissue. The potential due to the mirrored point sources is added to the potential due to the point-current source with a weight W_TS_. The weight is dependent on the conductivity of tissue σ_T_ and saline σ_S_, such that W_TS_ = (σ_T_ − σ_S_)/(σ_T_ + σ_S_*)*. Since σ_T_ is less than σ_S_, W_TS_^n^ in the first term and all odd terms (n = 1, 3, 5, …) is negative. At even iterations (n = 2, 4, 6, etc.), W_TS_^n^ is positive. In the first iteration, the potential due to mirrored point sources is subtracted from the potential of the main point source. Interestingly, the resulting potential-spatial-decay curve sharpens, as the potential surrounding the peak gets significantly reduced (see Supplementary Fig. S05b). This effect leads to reduced background activity from active neurons >150 μm away from the recording electrode. As an example, we applied MoI and simulated two point sources, representing two neurons within a tissue at 150 μm lateral distance from each other and both at a z-distance of 30 μm from the array. Assuming an electrode being directly below one neuron, hereon termed neuron A, we calculated the SNR at this electrode considering only one other neuron, neuron B, as the source of background noise. At 150 μm lateral distance, the measured potential at the electrode originating from neuron B is approximately 20% of the peak potential in a semi-infinite tissue, while this value is reduced to 14% in a 200 μm-thick slice. In this simplified computation, in which the noise only comes from background activity (at 150 μm lateral distance), the SNR for a 200 μm-thick slice (double boundaries) is approximately 40% better than the SNR for a semi-infinite tissue (single boundary).

#### Performance of different HD-MEA models for z localization

**Figure 6:**
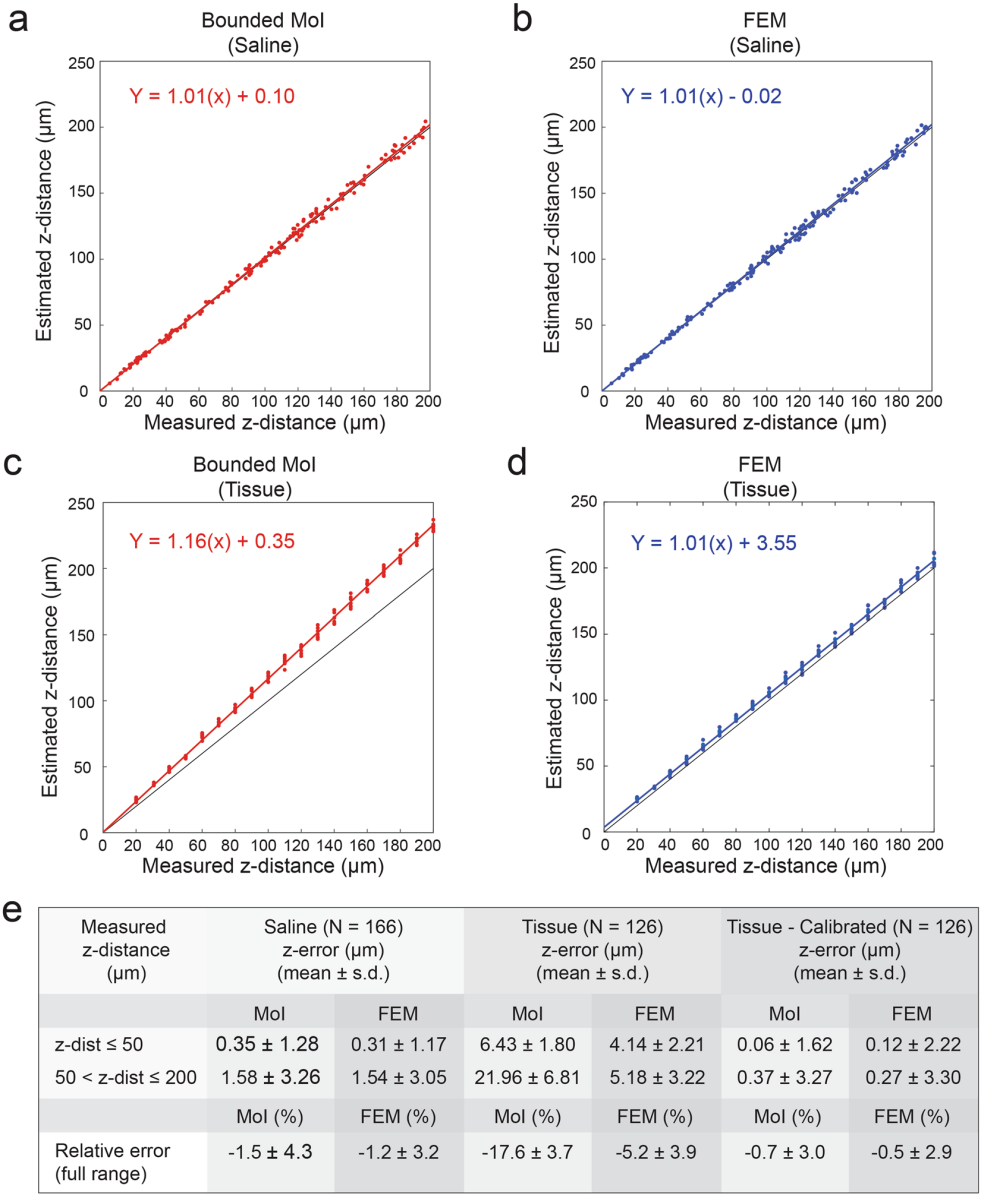
Localization performance along the z-axis. Accurate z-localization of a point source in saline N = 166 (**a**,**b**) and in an acute brain slice N = 126 (**c**,**d**) can be achieved using HD-MEA models. For each measurement, the estimated z-distance of the pipette is plotted against the measured z-distance. Estimates were based on the MoI model (red dots in **a** and **c**) and FEM (blue dots in **b** and **d**). The measured values refer to the programmed movement of the micromanipulator, which was confirmed visually by microscopy when possible. In (**c**,**d**), the location of the micropipette could not be monitored by microscopy so that only the micromanipulator movement was used as reference after a calibration step. Linear regression analysis was performed for both saline and tissue z-localization results for using MoI (red line) and FEM (blue line) models. The fitted regression lines indicate that FEM-based estimates are closer to measurement values as compared to MoI estimates. (**e**) Summary of z-errors computed by subtracting the measured z-distances (manipulator data, microscopy) from the estimated z-distances (FEM and MoI models). The mean values and standard deviations are shown.

We applied the least-squares fitting method to estimate the z-distance using the model-based potential spatial decay curves. The z localization algorithm is discussed in the Methods section. The results for the MoI model are shown in Fig. 6a (saline) and **c** (acute slice), while the results for FEM are displayed in Fig. 6b (saline) and **d** (acute slice). We plotted the estimated z-distances against the measured z-distances. Moreover, we performed a simple linear regression analysis (least squares) on the datasets, and the fitted regression lines demonstrate that MoI and FEM-based estimates were comparable for the saline-only case, whereas FEM-based estimates were closer to the measurement values in the presence of an acute brain slice.

A summary of the accuracy of the z-localization method is shown in Fig. 6e (mean ± s.d. of the difference between estimated and measured z-distances). For z-distances below 50 μm, the results for saline are 0.35 ± 1.28 μm and 0.31 ± 1.17 μm for MoI and FEM models, respectively. In the brain slices, the z-errors are 6.43 ± 1.80 μm for MoI and 4.14 ± 2.21 μm for FEM. For z-distances above 50 μm, the results are similar for both models in saline; the MoI and FEM z-errors are 1.58 ± 3.26 μm and 1.54 ± 3.05 μm, respectively. In tissue, the errors increased, and we obtained 21.96 ± 6.81 μm for MoI and 5.18 ± 3.22 μm for FEM. From the tissue results, we found that the systematic errors for both, MoI and FEM models are significant, however the values obtained with the FEM model were closer to the experimental results as compared to the MoI model. Thus, a pre-calibration step is recommended for z-localization using these models. We show the errors after the pre-calibration in Fig. 6e, last column. In summary, for z-distances considered in this study, the relative systematic error after pre-calibration is less than 1%, and the relative random error amounted to 3% for both, MoI and FEM. With 95% confidence, the z-localization predictions by both models will lie within ±4.4 μm from the expected value for z-distances below 50 μm and within ±6.6 μm for z-distances above 50 μm. The measured z-distances in tissue were based on micromanipulator movements only, so that it could be possible that the calibrated micromanipulator readings were off by a constant value. The linear regression analysis of the FEM (tissue) results indeed exhibited an approximate constant offset of 4 μm in the estimated z-distances between 5 to 200 μm. Nevertheless, z localization with a precision of <2 μm (standard deviation) for z-distances below 50 μm is sufficient for advanced electrophysiology experiments in acute slices. Such experiments may include combined patch-clamp and extracellular recordings or electroporation of extracellularly detected cells, as will be shown in the next section.

### Localization enables electroporation of spontaneously active cells

We used the localization method to augment acute-brain-slice extracellular electrophysiology. As mentioned in the introduction, subcellular resolution analysis of neuronal and neuronal network function can be achieved through HD-MEAs. To advance this technique for elucidating the function of single neurons, such as dendritic/axonal computation and the role of various genes in development, it is necessary to image a single neuron and to be able to locally deliver genes and plasmids to the same neuron, while recording from it. Electroporation has been used to efficiently transfect single cells for different applications, e.g., to deliver calcium indicators, dyes and activity modulators, such as channelrhodopsin-2, or ChR2^42–44^, to knock down gene expression via RNA interference^45,46^, or to trace monosynaptic inputs in neuronal networks^47^. A common single-cell electroporation protocol involves filling a micropipette with a plasmid solution, placing the micropipette close to the cell membrane, and stimulating with large voltage pulses (>5 V)^48^. Performing this procedure manually on neurons during HD-MEA recording requires skills and time.

**Figure 7:**
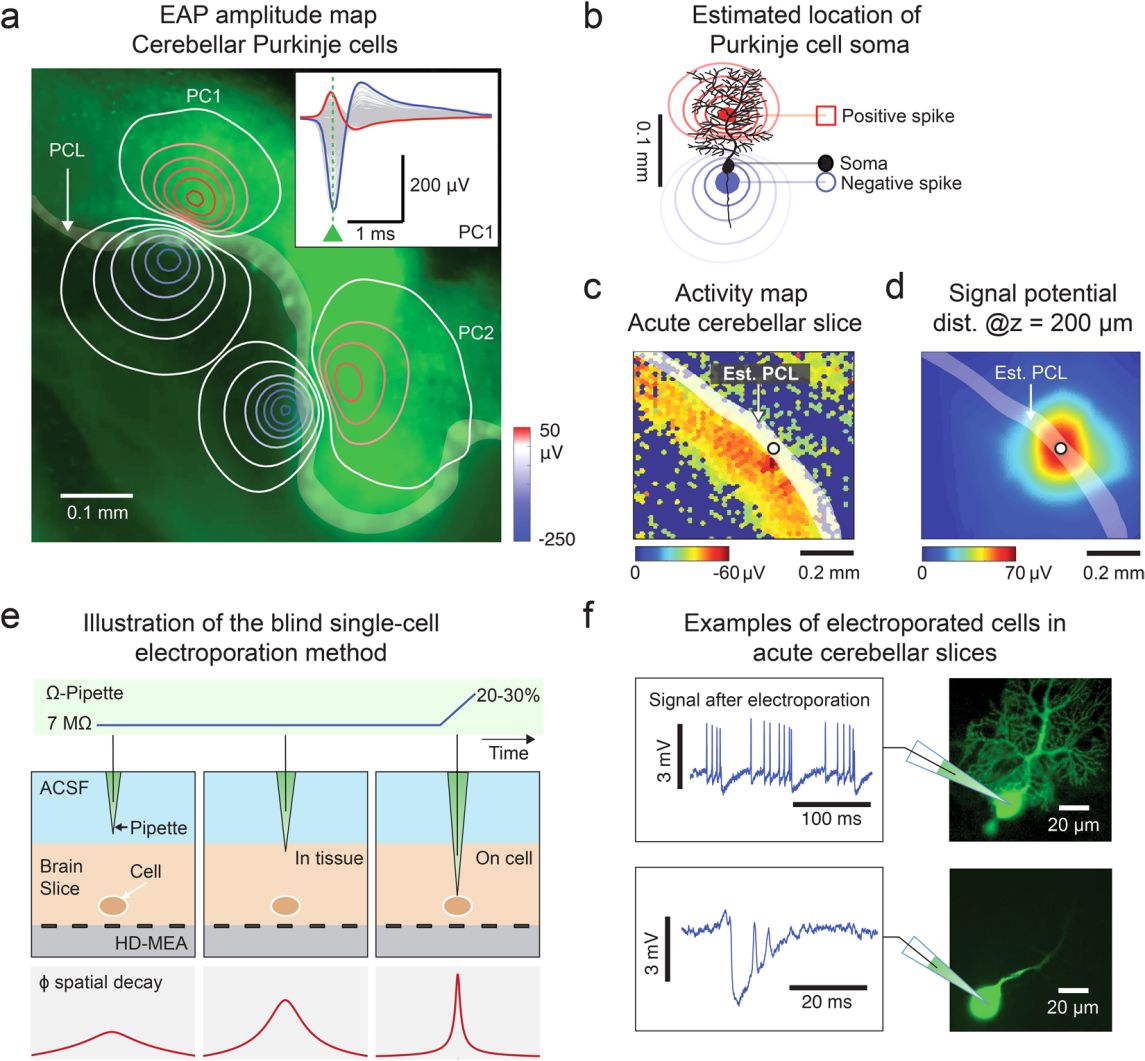
Application of the localization technique to target active neurons on the HD-MEA. (**a**–**d**) Experiments in the Purkinje cell layer (PCL) of an acute cerebellar slice, where Purkinje cell (PC) somas are located. (**a**) Extracellular action-potential (EAP)-amplitude map of two Purkinje cells (PCs), PC1 and PC2. The amplitude values were determined according to the largest negative peak of the EAP as indicated for PC1 by the green triangle in the inset. A superposition with a fluorescence-microscopy image of the cerebellar slice (GAD67 − GFP+) shows the correspondence between the EAP amplitude map and the Purkinje cell layer (PCL). (**b**) Cartoon illustrating the location of the soma based on the EAP amplitude map. The largest negative spike was detected at small distance to the soma and estimated to be at the axonal initial segment. (**c**) Activity map based on the negative EAPs obtained from spontaneously spiking PCs in an acute brain slice. The PCL was estimated to be on the right/upper side of the area with high-amplitude negative spikes as indicated. (**d**) Spatial electrical potential distribution generated by a stimulating micropipette *in tissue* (z = 200 μm). The white dot in c and d indicates the x-y location of the micropipette. (**e**) Sketch of the blind single-cell electroporation method. The pipette was filled with Lucifer yellow, a GFP dye, to visualize the electroporation results. As the pipette penetrated the tissue (*in tissue*), the potential detected by the HD-MEA increased drastically, despite a change in z-distance of only less than 20 μm. The pipette was moved ‘blindly’ within the brain slice until the pipette tip impedance increased, which indicated the presence of a cell membrane close to the pipette tip. The pipette was considered to be *on-cell*, when the tip impedance increased by 20–30% with respect to the value measured while the pipette was in the bath. Upon reaching *on-cell* state, the electroporation stimulation protocol was performed. (**f**) Examples of successfully electroporated cells. The electrical signals, acquired with the pipette after electroporation, reflected the success of the poration (left panel). The success of the electroporation was also evaluated based on the GFP staining quality, as visualized by fluorescence microscopy (right panel). Intracellular-like signals detected by the pipette mostly correlated with successful staining of the cells.

We used the localization method to guide a micropipette towards active neurons on an HD-MEA for electroporation. To approximate the position of the soma of active neurons, we analyzed HD-MEA extracellular recordings. We extracted the EAP amplitude map of two spontaneously active Purkinje cells in an acute cerebellar slice shown in Fig. 7a. The EAPs were obtained by spike-triggered averaging over 600 spikes, triggered at the electrode with the largest spike amplitude, using a threshold of 9 times the baseline noise level (calculated as the root mean square or RMS of the voltage)^11^. In parasagittal cerebellar slices, the Purkinje cells are the most prominent actively spiking cells and are aligned along the Purkinje cell layer (PCL). We correlated the EAP amplitude maps with the fluorescence microscopy image of the acute cerebellar slice, wherein most of the Purkinje cells were GFP positive (GAD67 − GFP+). From our results, we noticed that the largest negative spike was located at a few micrometers below the PCL (Fig. 7b), suggesting the source to be the axonal initial segment^49^. With this finding, we were able to estimate the location of the PCL based on the obtained activity map across an acute cerebellar slice. An example activity map shown in Fig. 7c consists of the mean of the largest negative spikes, i.e., the top 90% in terms of amplitude detected per electrode.

We targeted areas in the activity map with large spike amplitudes, so-called *areas of interest*. We blindly navigated the micropipette by using HD-MEA assisted localization to then electroporate cells. We localized the (x,y,z) position of the micropipette by issuing current signals through the pipette and analyzing the produced HD-MEA signals. A sample potential distribution plot used for localization is shown in Fig. 7d. While the pipette was still in saline above the slice, we used a high-amplitude signal for localization (50 nA, 1000 Hz). When the pipette reached a z-distance of 200 μm, we switched to low-amplitude pulses in voltage-clamp mode (50 mVpp, 1000 Hz. The same signal was used to monitor the impedance changes at the pipette tip. By delivering constant voltage pulses of 50 mV peak-to-peak amplitude, the current measured by the patch amplifier depended on the impedance of the pipette tip. Thus, as the pipette tip touched a cell membrane, the impedance change was detected as a reduction in the patch amplifier current readout.

For this proof-of-concept work, we aimed at approaching individual spontaneously spiking neurons in the PCL. Above 250 μm, we navigated the pipette in the x-y direction until it reached an area of interest. Once the pipette was detected by the HD-MEA to be in the area of interest (large amplitude spikes), we moved the pipette down to the tissue until the pipette reached the cells. We also estimated the z-distance of the pipette within the tissue to prevent the pipette from touching the array surface and to target cells within 70 μm distance from the array. We monitored the signal detected at the pipette tip through the patch-clamp software (PClamp), while the micropipette approached the cell membrane of a target neuron and stopped the approach when a 20–30% change in impedance was observed. The 20–30% increase in pipette impedance indicated that the pipette was *on-cell*. We then checked whether spontaneous extracellular spikes could be detected by the micropipette and only proceeded with the electroporation if spontaneous spikes were observed. Otherwise, we retracted the pipette tip and searched for another cell. We stopped every trial at a minimum z-distance of 30 μm and retracted the pipette back up to a z-distance of 250 μm to start a new approach if no cell was found (no change in pipette tip impedance). A summary of the electroporation method is illustrated in Fig. 7e, and an example case is shown in Supplementary Fig. S07.

We performed electroporation by stimulating the micropipette with current pulses between 0.5 and 2 s (100 nA, 200 Hz). Since we only electroporated spiking cells, we assumed that we most likely approached cerebellar Purkinje cells. We visually checked the electroporated cells afterwards by using fluorescence microscopy, and all the stained neurons exhibited morphologies that were typical for Purkinje cells. We assessed the electroporation and staining quality and also evaluated the response of the neuron to electrical stimulation. Examples of electroporated cells can be seen in Fig. 7f. Electroporation was considered successful if intracellular signals could be observed through the micropipette immediately after issuing stimulation signals by means of the micropipette (7 out of 11 cells). Cells that exhibited intracellular and intracellular-like signals (large positive spikes) usually were stained well with the dendrites being clearly visible and distinguishable. In the cases that we could only record extracellular spikes or no spikes through the micropipette after electroporation, the electroporation was considered unsuccessful, and the cells also turned out to not have been stained properly upon subsequent inspection by means of a fluorescence microscope (4 out of 11 cells).

## Discussion

We have shown that blind 3D localization of micropipettes can be done using HD-MEAs. To realize such localization, we used 2D Gaussian fitting for the x-y localization. While a Gaussian curve is neither equal to MoI (Equation 2), nor FEM-based potential spatial decay curves, it proved to be suited for accurate position estimates, as we only needed to find the peak of the potential distribution. Due to the large number of measurement electrodes, located at precisely defined locations that are defined by the lithographic process, the resulting random error was significantly below 1 μm, which is much less than the center-to-center pitch of the electrodes. The x-y localization method was found to be precise within ±2 μm, with 95% confidence, for z-distances up to 200 μm in both, saline and tissue samples.

For the z localization, we devised HD-MEA models based on MoI and FEM. We compared the potential spatial decay curves, extracted from the models, to the decay curves obtained from experiments. The MoI model provided an approximation of the HD-MEA point spread function, however, FEM results proved to better match the experimental data, especially for z-distances above 50 μm. In summary, results obtained with both, MoI and FEM models were comparable to experimental results in saline with relative systematic errors amounting to −1.5% and −1.2% for MoI and FEM, respectively. Similarly, both models provided precise z-localization results, with relative random errors <5%. With 95% confidence, the z-localization predictions by both models will lie within ±2.6 μm from the expected value for z-distances below 50 μm and within ±6.6 μm for z-distances higher than 50 μm. For the tissue case, the FEM-model results with −5.2% relative systematic error, were closer to the measurement values compared to −17.6% systematic error of MoI. Thus, a pre-calibration step is recommended for both models, so that they can be used for accurate z-localization. After pre-calibration, the systematic errors were significantly reduced to −0.7% and −0.5% for MoI and FEM, respectively. In terms of precision, the relative random errors were also below 5% for both models. After pre-calibration, the precision of both, MoI and FEM for z-localization in tissue became comparable. The localization results were within ±4.4 μm for z-distances below 50 μm, and ±6.6 μm for distances above 50 μm with 95% confidence. These results show that the method proposed here is sufficiently accurate to target cell somas with typical diameters of 10 to 30 μm.

Using the models, we also quantified different factors shaping the electrical field on HD-MEAs. Insulating boundaries in the model were found to increase the amplitude of the signal detected by the HD-MEA: the presence of an insulator HD-MEA surface doubled the magnitude of the potential, and the presence of a liquid-air interface increased the magnitude further, approximated by an added constant value for liquid heights above 1 mm. A similar effect could be attained through an additional insulating plane close to the HD-MEA surface, such as a resistive sheet of glial cells for cell cultures^23^, axonal tunnels^24–26^, a layer of mineral oil or a glass slide on top of the preparation. The potential increase can be approximated by using a bounded MoI (Equation 3).

The effects of grounding constitute another factor that influences the potential-spatial-decay curves. The grounding effect more and more decreases the measured potential upon approaching the actual location of the ground structure. We illustrate this effect in Supplementary Fig. S04. For our experiments, we placed the ground at the chamber ring, which was also included in the FEM models. To include the grounding effect in MoI models, it is possible to compute the potential at the ring radius (*ϕ*_ring_ = 8.5 mm) and subtract the obtained constant value from the potential across the spatial extension of the bounded MoI model. We tested this analytical solution for the potential spatial distribution of a point-source at a z-distance of 50 μm. The resulting potential distribution was similar to that obtained with FEM, (see Fig. 3a). Subtracting a constant value, equivalent to *ϕ*_ring_, works when the point source is close to the center of the chamber, and the ground is equidistantly far away. However, if the ground is closer to the electrode array, the grounding effect will be shaped depending on the location of the point source with respect to the ground. In this case, the grounding effect is no more constant across the potential decay curve, if the point source is, e.g., located at the corner of the electrode array.

For the localization of neural activity, MoI will work well even without considering the grounding effect. Due to current conservation laws, the grounding effect is, in this case, negligible. The potential distribution of extracellular action potentials is localized, so that, e.g., in the case of a dipole, the potential decays quickly unlike the potential of a point source. Any neuron, irrespective of its location with respect to ground, can, therefore, be localized accurately using MoI.

For HD-MEA recording of neuronal signals, the signal-to-noise ratio (SNR) is an important factor for identifying and discriminating active units^50,51^. Insulating boundaries increase the electrical potential, detected by the array, and improve the detection of very small signals but at the expenses of increasing background noise. However, the effect of the tissue-to-saline boundary is different. The “focusing effect” potentially improves the localization of a signal within a tissue slice, as detected by the HD-MEA.

In our localization experiments, we kept the signal source fixed at a certain position, while we recorded for 10 s. We used high enough current stimulation amplitudes (50 nA, 1 kHz), to have sufficient SNR for pipette localization. The major noise sources in HD-MEA recordings include the electrodes and the amplification read-out chain. We measured a noise level of 4.7 μV_rms_ (AP band: 300–3000 Hz) by making recordings in the same experimental HD-MEA setup without any signal source, however, in the presence of a micropipette in the HD-MEA dish. Depending on the localization experiment, the integration time can be adjusted so that a longer integration time would yield more accurate results, whereas a shorter integration time would enable faster feedback for live tracking purposes. In our tissue experiments, the applied stimulation amplitude and frequency had no perceivable effects on the viability of spontaneously spiking cells during the localization procedure.

The localization method can be applied to control moving micropipettes for advanced electrophysiology experiments by combining HD-MEA recordings with single-cell-targeted experiments, such as local puffing of compounds^52–54^, electrically-guided automated intracellular recordings^30–32,55^, virus-stamping^56^, single-cell electroporation^44–46^, and other single-cell-based methods. Other advanced single-cell experiments, such as Patch-seq, can be easily implemented together with HD-MEA extracellular recording. Patch-seq involves whole-cell patch-clamp recordings, single-cell RNA sequencing, and morphological cell characterization^57^. Such combination techniques will enable a detailed analysis of single cells in functional neuronal networks.

Moreover, combining HD-MEAs with pipette-based dye-loading^58^ or single-cell electroporation allows for obtaining morphologies of recorded cells on the HD-MEAs. This way, comprehensive information can be acquired for developing precise and realistic multi-compartment models. The density of membrane current sources in a neuron can be computationally approached through the extracellular potential landscape by using a method called “current-source-density (CSD)” analysis^59,60^ (see reviews^61,62^). If the shape or morphology of a neuron is given, the membrane currents of the neuron can be estimated by backward estimation based on spatial EAPs obtained from HD-MEA recordings^14,21,63^. Realistic neuronal models provide insights on the function of the cell and facilitate the identification and localization of specific neuronal types in HD-MEA experiments *in vitro* and *in vivo*^16–18^. The developed technique may also find applications outside the field of electrophysiology, for localizing and guiding an electrically conducting point source inside a volume conductor without using optical means.

## Materials and Methods

### HD-MEA

We used a CMOS-based high-density microelectrode array (HD-MEA)^1,14^ for localization experiments and extracellular neuronal recordings (Supplementary Fig. S01). The HD-MEA features 11,011 electrodes in an area of 1.99 × 1.75 mm^2^ (17.8 μm center-to-center pitch, 3′161 electrodes/mm^2^ density). The size of each platinum electrode size is 10.2 × 8.6 μm^2^. Platinum black was deposited on the electrodes to lower their impedance. Any subset of electrodes could be flexibly routed to 126 channels for recording. The sampling rate per channel was 20 kS/s at 8-bit resolution. All recorded signals were amplified 1450-fold and filtered (high-pass: ~4 Hz, low-pass: 3.7 kHz) using on-chip circuitry. We characterized the relative gain variability (at 1 kHz) across all electrodes for each HD-MEA, which was approximately ~5%. In order to compensate for this effect in all localization experiments, we multiplied the recorded signals with a compensation factor (determined earlier for each electrode) offline before data analysis. To accommodate micropipette stimulation and upright microscopy, we used HD-MEAs with 4 mm and 8 mm chamber heights. An external platinum wire attached to the inner wall of the HD-MEA chamber served as the reference electrode.

### Solutions

All chemicals were obtained from Wako Chemicals (Japan) and Sigma (St. Louis, MO). *Saline solution:* PBS tablet dissolved in 200 ml of MilliQ purified water, which yielded 10 mM phosphate buffer, 2.7 mM KCl, and 137 mM NaCl. *Standard artificial cerebrospinal fluid (ACSF):* 125 mM NaCl, 2.5 mM KCl, 1.25 mM NaH_2_PO_4_, 1.9 mM MgSO_4_, 20 mM Glucose, 25 mM NaHCO_3_, 2 mM CaCl_2_. *Dissection ACSF:* standard ACSF without CaCl_2_.

### Acute slice preparation and recording

All animals were obtained from the Laboratory for Animal Resources and Genetic Engineering at RIKEN Center for Developmental Biology in Kobe, Japan. All experimental procedures were approved by the local authorities (Animal Care and Use Committee of RIKEN; QAH24-01) and executed in accordance to RIKEN Guidelines.

Adolescent wild-type CD-1 and GAD67-eGFP^36^ mice (P18 to P35) were deeply anaesthetized by isoflurane inhalation and then decapitated. Brains were dissected out and immediately immersed in ice-cold dissection ACSF. Cerebellar tissues were harvested. The cerebellum was glued onto the vibratome tray along its sagittal plane. The tissues were kept in ice-cold dissection ACSF, bubbled with carbogen (95% O_2_ and 5% CO_2_) during slicing. Parasagittal cerebellar slices (200 μm thick) were cut using a Leica vibratome VT-1200S. The slices were transferred to carbogen-bubbled warm ACSF (35 °C) and were allowed to recover for at least 40 minutes before placement on the HD-MEA.

Slices were carefully positioned flat on the HD-MEA surface for recording. A custom-made weight kept the slices in place. Slices were superfused with carbogen-bubbled standard ACSF at 36 °C. Spontaneous electrical activity detected by the HD-MEA in this setup persisted up to 6 hours after incubation.

### Localization experiments

#### Experimental set-up

Borosilicate glass micropipettes with filaments were pulled using a P-97 pipette puller (Sutter Instruments, Novarto CA, USA) to have tip resistances between 5–7 MΩ. The tip resistance and output current were monitored with the Clampex software (Molecular Devices, Sunnyvale, USA). A glass micropipette was filled with either saline solution or standard ACSF, connected to a patch amplifier (MultiClamp 700B, Molecular Devices, Sunnyvale, USA), and mounted on a micromanipulator (Patch-star, Scientifica, East Sussex, UK). The patch amplifier controlled the stimulation amplitude. The pipette was positioned atop the HD-MEA by using the micromanipulator, and its distance from the array surface was confirmed using a microscope (Olympus BX61 with a 40x water immersion objective). A schematic of the setup is shown in Supplementary Fig. S02. The micromanipulator software (LinLab, Scientifica, East Sussex, UK) was used to control the micropipette movement at 0.25 μm precision. The micropipette was stimulated with a sine- or square-wave featuring a peak-to-peak output current of 50 nA at 1 kHz. The signals were recorded using the HD-MEA.

#### Amplitude extraction

We extracted the magnitude of the potential, detected by each electrode, from the discrete Fourier transform (DFT) of the recorded signal, computed using a fast Fourier transform algorithm in Matlab R2015b (FFTW library). We obtained the peak voltage between 990 to 1010 Hz from the single-sided amplitude spectrum. For square-wave signals, we only extracted the first harmonic and divided the amplitude by 2/π to get an equivalent value to sine-wave stimulation.

#### Localization algorithm

For simplicity, we separated the localization problem into two parts: x-y localization and z localization. In this way, we were able to extract the x-y location of the point source independently of its z location.

x-y localization:In search of the location of the peak amplitude, we fitted the measured potential values to a standard 2D Gaussian distribution of the form:

$$f(x,y)=A\,\exp (-\,\frac{{(x-{x}_{0})}^{2}}{2{\delta }_{x}^{2}}-\frac{{(y-{y}_{0})}^{2}}{2{\delta }_{y}^{2}}),$$

where *A* was the amplitude of the distribution, (*x*_0_, *y*_0_) was the center of the distribution or the estimated x-y location of the micropipette, and (*δ*_x_, *δ*_y_) were the standard deviations that define the width of the ‘bell curve’. We used least-squares curve fitting (lsqcurvefit function in Matlab R2015b). The Gaussian curve was only used to find the peak irrespective of the z-distance of the point source.

z localization:We simulated the potential spatial decay curves using different models (semi-infinite MoI for saline, bounded MoI for acute slice, and FEM) for z-distances between 1 and 1000 μm, with a step size of 1 μm. We limited our search to 1000 μm z-distance, as all measurements were only conducted up to this z-distance. We searched for the closest spatial decay curve fit per measurement by applying the least squares method. For each experiment, we first converted the measured potential values to a potential spatial decay curve. We used the estimated x-y location as the peak of the curve, where the radial distance is zero. Then, we computed the sum of squared residuals (*S*_*z*_) between a single experimental decay curve and each of the model decay curves for all z-distances. The z-distance was estimated to be at z where *S*_*z*_ is closest to zero.

### Modeling HD-MEA signals

We modeled the HD-MEA signals by: (i) applying method of images (MoI) to volume conductor theory and (ii) using a finite-element method (FEM). The basic principles regarding the electric field of a signal source in a conductive volume is well established^27^. Properties of the extracellular space shape the electric field also in the electrode-array plane. Assuming an infinitely high input impedance of the amplifier circuits and point–like electrodes, the electric potential in the liquid phase can be calculated independently of the electrode properties. In this work, we focus on the signal shaping from the signal source to the recording electrode.

#### Volume conductor theory

At the frequency bands considered for neuronal activity detection (<5000 Hz), the brain tissue can be considered as purely Ohmic (i.e., no capacitive component) and the transmission of signals in the tissue can be modeled by using quasi-static Maxwell equations^64–66^. The volume conductor regime in an HD-MEA experiment refers to the passive region above the electrode array (e.g., saline, tissue, or culture media) where the electric field is present. To analyze the signal of a point current source that is recorded by a point electrode, volume conductor theory can be applied^27,67,68^. The potential *ϕ*, recorded at an electrode located at a distance *r′* = (*x′*, *y*,′ *z′*) from a point current source *I*, located at *r* = (*x*, *y*, *z*), assuming an infinite, homogeneous, and isotropic volume conductor with conductivity *σ*, is given by:

$${\rm{\varphi }}({\rm{x}}^{\prime} ,{\rm{y}}^{\prime} ,{\rm{z}}^{\prime} )=\frac{{\rm{I}}}{4{\rm{\pi }}{\rm{\sigma }}\sqrt{{({\rm{x}}^{\prime} -{\rm{x}})}^{2}+{({\rm{y}}^{\prime} -{\rm{y}})}^{2}+{({\rm{z}}^{\prime} -{\rm{z}})}^{2}}}.$$

*In vitro* experiments using HD-MEAs require an extension of this equation to include the electrode-array plane. Moreover, the conductivity distribution in the brain tissue, including properties such as anisotropy and inhomogeneity, and the boundaries at the interfaces (e.g. tissue to saline, saline to air) need to be considered for a more accurate model.

#### Semi-infinite MoI model

MoI provides an analytical solution to the potential in a volume conductor involving boundary surfaces, which apply to HD-MEA measurements^19–22,35^. Inhomogeneity of the conductor in the *z*-direction can be introduced to the volume conductor equation for *ϕ* using MoI. The HD-MEA surface and the liquid-to-air interface cause inhomogeneity in the *z*-direction. The interfaces between different conductors are considered as boundaries.

MoI replaces the boundaries with virtual point current sources, located at appropriate positions, to meet the respective boundary conditions. As an example, an equivalent solution to the effect of an infinite insulating plane on the potential at point *r* due to a point current source at point *r′* is to simulate an identical point current source, positioned at exactly the same distance on the opposite side of the boundary, and to remove the insulator. The contribution of this virtual point current source to the potential at any point in the volume conductor is then scaled with the conductivity factor *W*_12_ = (*σ*_1_ − *σ*_2_)/(*σ*_1_ + *σ*_2_) in reference to the real source^19,69^. *σ*_1_ refers to the conductivity of the region, where the real point current source is; *σ*_2_ refers to that of the virtual point source. In the case where a point current source is in a homogeneous and isotropic medium atop an insulating plane (i.e., in a first-order assumption in saline on a HD-MEA), the conductivity at the insulator is zero (*σ*_2_ = 0, *W*_12_ = 1), and the potential at the electrode surface simply doubles compared to the infinite volume conductor situation. This is accounted for in the following equation:

$${\varphi }_{se}(x^{\prime} ,y^{\prime} ,0)=2\ast \varphi (x^{\prime} ,y^{\prime} ,0)$$

where *σ* = *σ*_1_.

#### Bounded MoI model

The semi-infinite MoI model can be further extended to consider two boundaries, in order to include the effects of the liquid-air boundary. For a point current source, located within two boundaries, where *h* is the height of the liquid, the following equation can be used:

$${\varphi }_{bo}(x^{\prime} ,y^{\prime} ,0)={\varphi }_{se}(x^{\prime} ,y^{\prime} ,0)+2\sum _{n=1}^{\infty }\,{W}_{AB}^{n}[\varphi (x^{\prime} ,y^{\prime} ,-\,2nh)+\varphi (x^{\prime} ,y^{\prime} ,2nh)]$$

with *W*_AB_ = (*σ*_A_ − *σ*_B_)/(*σ*_A_ + *σ*_B_), where *σ*_A_ is the conductivity of the saline and *σ*_B_ is zero (*W*_AB_ = 1).

All the above equations can also be converted to use line-sources instead of point sources^19^, in order to estimate transmembrane currents from neurons^21,28,70,71^.

#### Finite Element Method

FEM simulations were performed using COMSOL Multiphysics version 5.2 and COMSOL with Matlab. The FEM model used here included the insulating boundaries, i.e., the liquid-air interface, chamber wall, and HD-MEA surface. A reference electrode was also included, either placed along the inner chamber wall or as a separate rectangular frame electrode surrounding the electrode array area. Two tissue models were created, isotropic tissue and anisotropic cerebellar tissue (see Supplementary Fig. S03 for more details). The point source was placed at different locations atop the electrode array. The electrode array was modeled as an insulator.

#### Electrode Size

We modeled the spatial averaging effect due to electrode size by modeling *ϕ* at 100 random points within an electrode area, centered around specific x′, y′ locations, and then obtained the average of all *ϕ*-values for each simulated electrode.

### Accuracy

We used the terminology *accuracy* as a general parameter to characterize the quality of the localization method, as it describes the closeness of a measurement to the true value while it includes components of (i) systematic error and (ii) random error. The systematic error is reflected in the *trueness* of the localization result. We used the mean error or a relative measure, the mean percentage error (MPE) to describe the trueness of the localization method. The MPE is the computed average of relative errors in percent, by which the results, obtained with the models, differ from experimental values. The equation for MPE is as follows:

$$MPE=\frac{100 \% }{n}\sum _{t=1}^{n}\frac{{e}_{t}-{m}_{t}}{{e}_{t}}$$

where *e*_*t*_ corresponds to an experimental data point, *m*_*t*_ is the data point obtained with either the MoI or FEM model, and *n* is the number of trials. The *precision*, on the other hand, indicates the random error of the localization results. We quantified the precision using the standard deviation (s.d) of the errors, both, in μm and as a relative measure in percent. To specify the prediction interval, we used a 95% confidence level, so that 95% of the readings were within the interval of mean value ± (2 × s.d.).

## Supplementary information

Supplementary Info and Figures

## Data Availability

The data sets and code generated during the current study are available from the corresponding author on reasonable request.
