# Author Correction: Accurate signal-source localization in brain slices by means of high-density microelectrode arrays

**DOI:** 10.1038/s41598-020-61780-y

**Published:** 2020-03-11

**Authors:** Marie Engelene J. Obien, Andreas Hierlemann, Urs Frey

**Affiliations:** 1 Department of Biosystems Science and Engineering, ETH Zurich, Basel, Switzerland; 2 RIKEN Quantitative Biology Center, Kobe, Japan; 3 MaxWell Biosystems AG, Basel, Switzerland

Correction to: *Scientific Reports* 10.1038/s41598-018-36895-y, published online 28 January 2019

The Acknowledgements section in this Article is incomplete.

“This work was supported by the Japan Society for Promotion of Science (JSPS) under Grants-in-Aid for Young Scientists (B) No. 25730183, the Swiss National Science Foundation through Grant 205321_157092/1 (“Axons”), as well as by the European Community through the European Research Council Advanced Grant 694829 ‘neuroXscales’. The funders had no role in study design, data collection and analysis, decision to publish, or preparation of the manuscript. We thank Alexander Stettler for post-processing CMOS chips and the D-BSSE support staff for help with experiments.”

should read:

“This work was supported by the Japan Society for Promotion of Science (JSPS) under Grants-in-Aid for Young Scientists (B) No. 25730183, the Swiss National Science Foundation through Grant 205321_157092/1 (“Axons”), as well as by the European Community through the European Research Council Advanced Grant 694829 ‘neuroXscales’ and through the Marie Skłodowska-Curie Individual Fellowships Grant 798836 ‘MAPSYNE’. The funders had no role in study design, data collection and analysis, decision to publish, or preparation of the manuscript. We thank Alexander Stettler for post-processing CMOS chips and the D-BSSE support staff for help with experiments.”

