# Supplementary material for: Accurate signal-source localization in brain slices by means of high-density microelectrode arrays
Source: Sci Rep. 2019 Jan 28;9:788. doi: 10.1038/s41598-018-36895-y (PMC6349853; doi:10.1038/s41598-018-36895-y)
Supplement: Supplementary file 1 — Supplementary Info and Figures [file 41598_2018_36895_MOESM1_ESM.pdf]

Accurate signal-source localization in brain slices by means of high-density microelectrode arrays

Marie Engelene J. Obien[1, 2, 3*], Andreas Hierlemann[1], Urs Frey[1, 2, 3]

[1]Department of Biosystems Science and Engineering, ETH Zurich, Basel, Switzerland

[2]RIKEN Quantitative Biology Center, Kobe, Japan

[3]MaxWell Biosystems AG, Basel, Switzerland

*** Correspondence:**
Marie Engelene J. Obien
marie.obien@bsse.ethz.ch

Keywords: signal localization[1], microelectrode array[2], micropipette[3], electroporation[4], extracellular recording[5], method of images[6], FEM[7], acute brain slice[8].

Supplementary Information

Equation for point source above two boundaries, MEA surface and tissue-saline interface

For situations when the point current source is above the slice, another approach for MoI includes to consider the tissue-saline interface. The equation where *z'* > *h* is (Jennings and Jones, 1988):

$$\phi_{bo,z>h_s}(x',y',0) = \phi_{se,\sigma_s}(x',y',0) + \gamma_{ST},$$

where

$\gamma_{ST} = 2\sum_{n=2}^{\infty} W_{ST}^{n}\left[\phi_{\sigma_s}(x',y',-2nh_s)\right]$, $W_{ST} = (\sigma_S - \sigma_T)/(\sigma_S + \sigma_T)$, and h_s is the height of the tissue.

Supplementary Figures

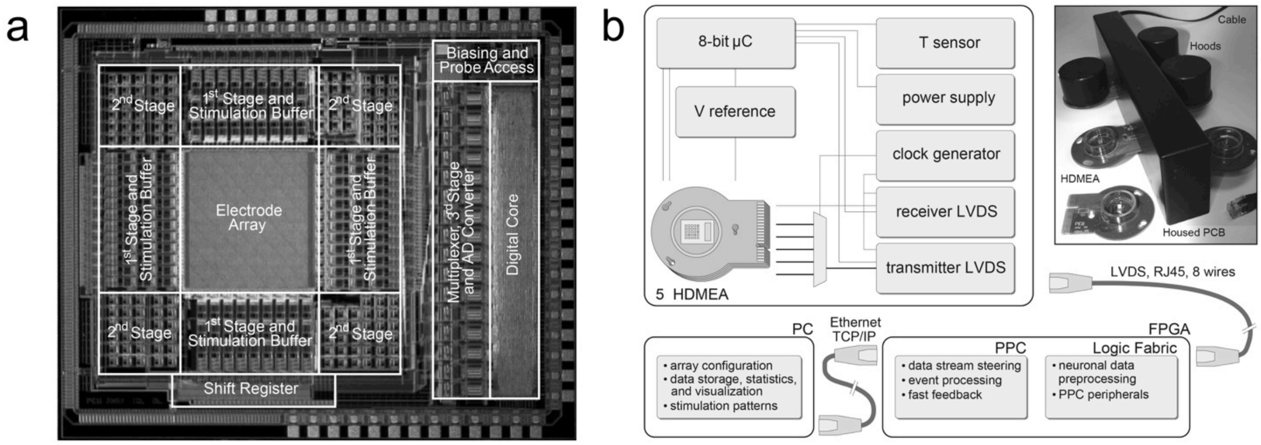

Supplementary Figure S01. Complementary metal oxide semiconductor or CMOS-based high-density microelectrode array (HD-MEA) used in this work. (**a**) Chip micrograph. The size of the chip is 7.5 × 6.1 mm^2, and the active area of the electrode array is 2.0 × 1.75 mm^2. (**b**) Block diagram of the HD-MEA setup and an example image of the acquisition unit with five HD-MEAs plugged in.

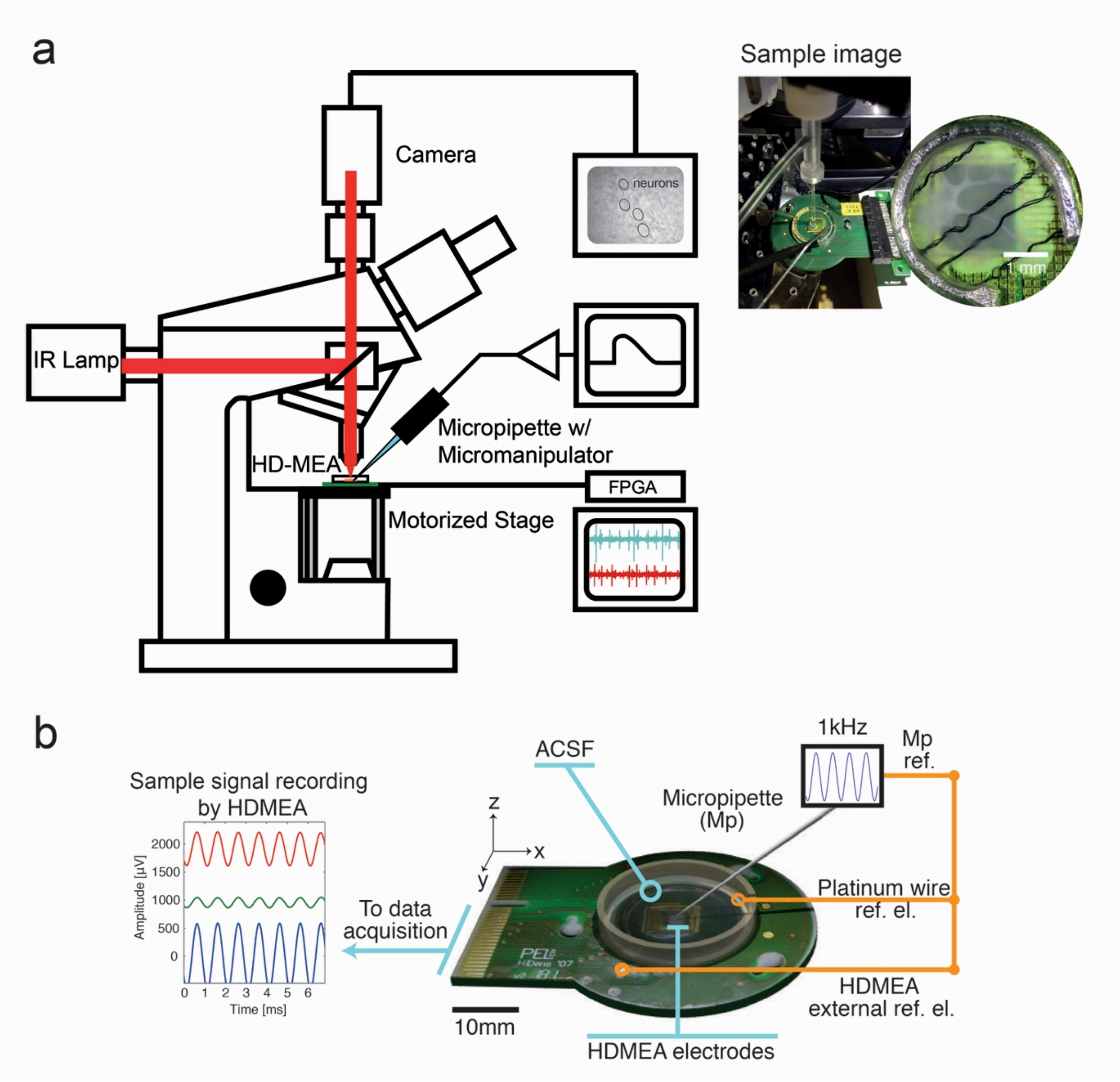

Supplementary Figure S02. Experimental setup. (**a**) Setup for obtaining the electrical potential distributions by means of an HD-MEA and for signal-source-localization experiments in saline and with an acute brain slice. The upright microscope is equipped with IR-DIC filters for better visualization of the micropipette tip on top of the HD-MEA. The HD-MEA is mounted on a motorized stage to precisely position the array under the microscope. The micropipette is controlled by a micromanipulator. The signals, detected by the micropipette, and the stimulation through the micropipette are controlled and visualized using a patch-clamp setup (Clampex software, MultiClamp amplifier, Digidata digitizer). The microscope was not used for experiments with an acute brain slice, as the micropipette was adjusted to approach the HD-MEA perpendicularly (inset). (**b**) Close-up of the HD-MEA chip. The connection of the reference electrode and the recording of signals upon micropipette stimulation by means of the HD-MEA are shown.

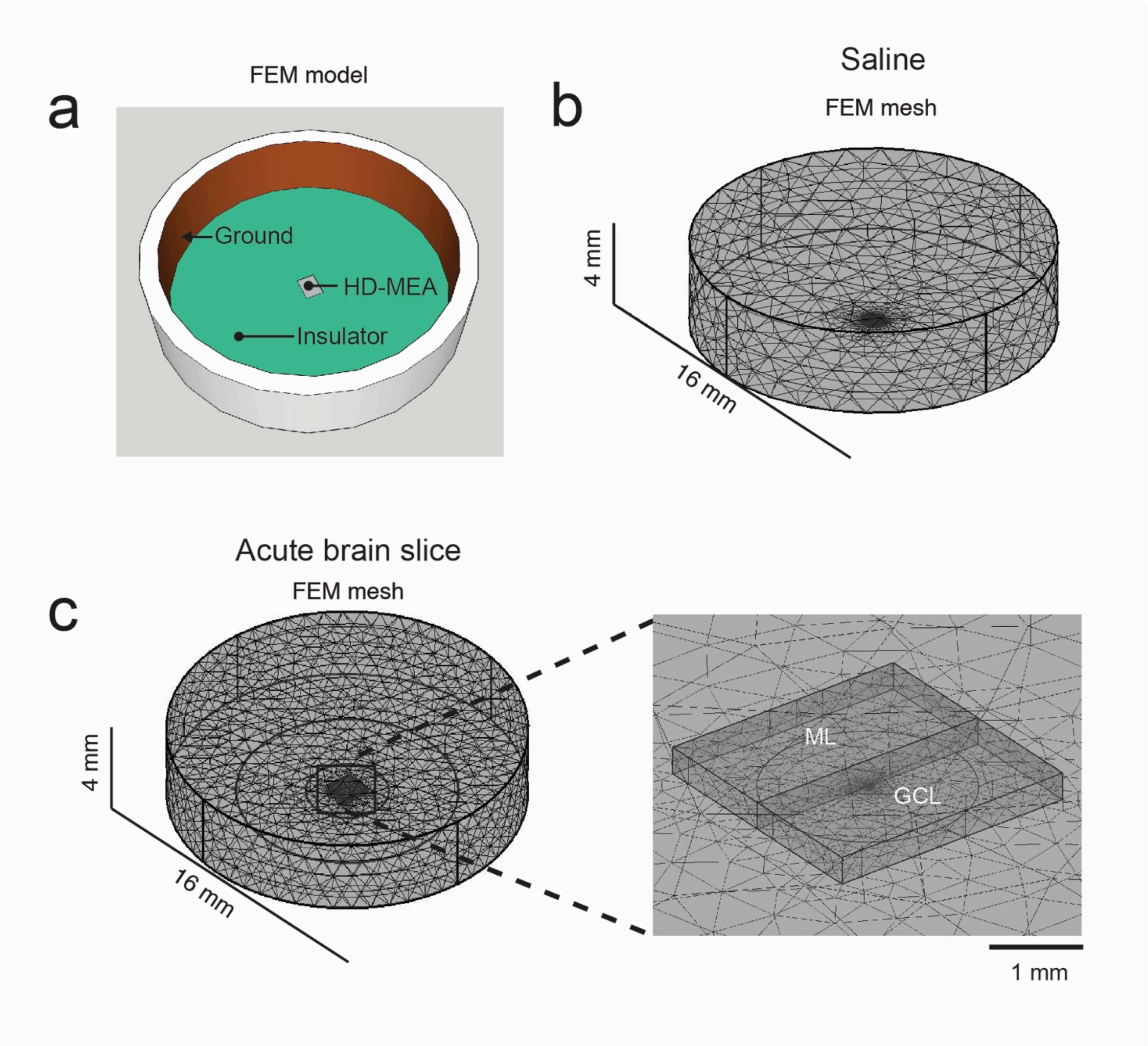

Supplementary Figure S03. Descriptive sketches of the FEM models. (**a**) Sketch of the HD-MEA FEM model. The bottom of the HD-MEA chamber is an insulator, the chamber wall served as the reference electrode, and the HD-MEA surface was modeled as both, an insulator and a conducting electrode connected to ground through a high impedance. The HD-MEA chamber is filled with a conducting liquid (saline, not shown). (**b**) FEM mesh for the saline case. (**c**) FEM mesh with an acute brain slice. The molecular layer (ML) and granular cell layer (GCL) of a cerebellar slice were modeled.

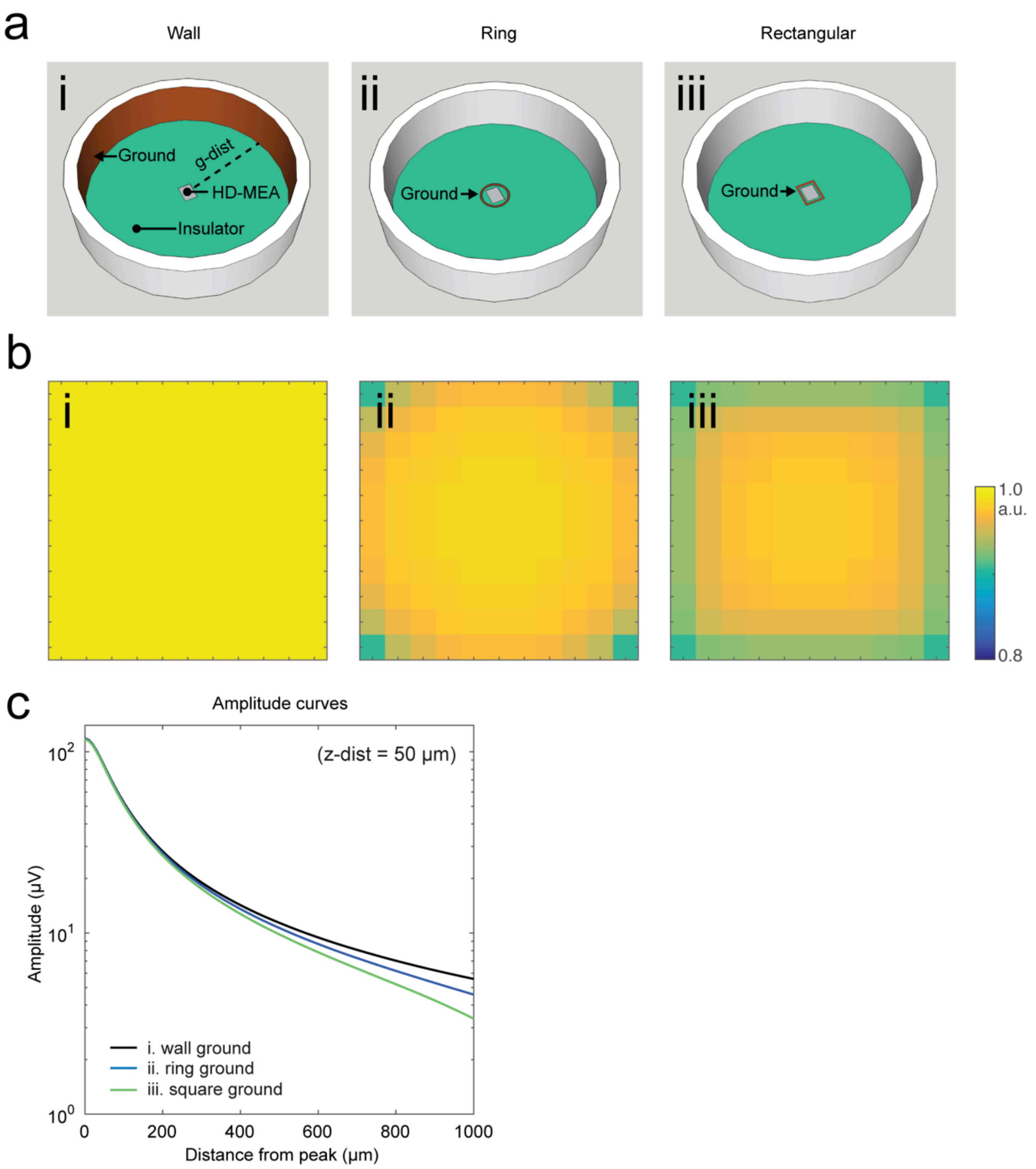

Supplementary Figure S04. Illustration of the 'grounding effect' using FEM. (**a**) Illustration of three different versions of grounding, (i): ground at the chamber wall, (ii): ring ground surrounding the electrode array, and (iii): square ground surrounding the electrode array. (**b**) The maximum amplitude is computed for each location on a grid (2×2 mm^2). All results in this figure are obtained from FEM simulations with the point source located at a z-distance of 50 μm. The point source is moved across a grid covering the electrode array, while keeping the z-distance at 50 μm. The relative amplitudes are plotted, which do not show any changes in maximum amplitude values when the ground is at the chamber wall, 8.5 mm away, while the maximum amplitude is reduced at the edges of the array, when the ground is only 0.2 mm away (ii and iii). (**c**) Amplitude curves obtained for different ground types, when the point-source is at the center of the array.

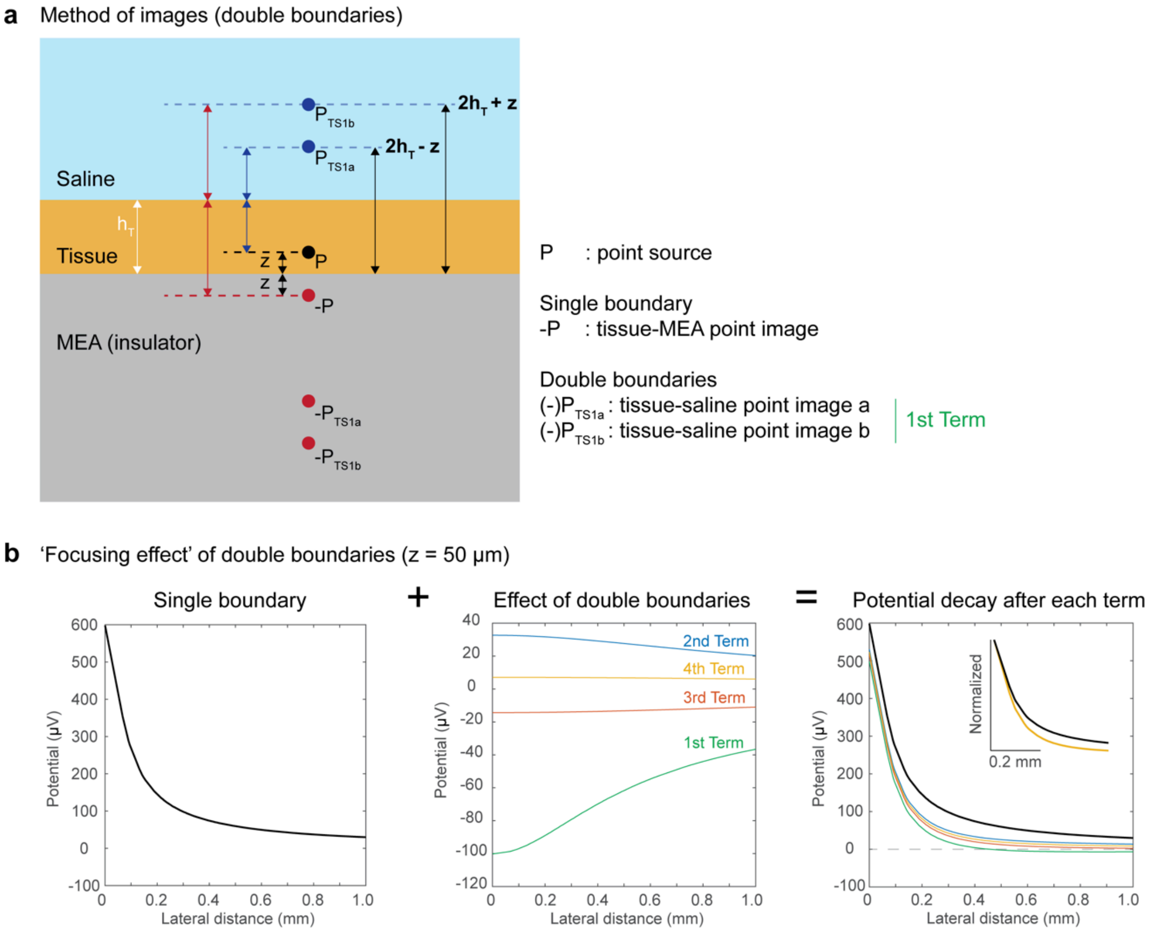

Supplementary Figure S05. Illustration of the brain tissue 'focusing effect'. The tissue-to-MEA interface serves as the first boundary and the tissue-to-saline boundary as the second. (**a**) Visualization of the bounded method of images (Equation 4), showing the point source and the equivalent mirrored point sources due to interface boundaries. The main contributor to the potential spatial decay is 2P (P – (-P)), located in the tissue at a distance z from the MEA surface. The potential spatial decay of 2P is equivalent to the single-boundary situation. The potential distributions of the mirrored point sources above the MEA surface that are due to a second boundary are added to 2P, thus sharpening the spatial decay curve. (**b**) Sample representation of the effect of double boundaries. (Left panel) Potential spatial decay for the single boundary case, equivalent to a semi-infinite tissue. (Middle panel) Potential spatial decay curves caused by the second boundary (tissue-to-saline). Each term to be summed in γ_{TS} (Equation 4), caused by mirrored point images, is weighted by W_{TS}. The weight is dependent on the conductivity of the tissue σ_T and saline σ_S, in that $W_{TS} = (\sigma_T - \sigma_S)/(\sigma_T + \sigma_S)$. Since σ_T is less than σ_S, $W_{TS}{}^n$ in the first iteration and for all odd terms (n = 1, 3, 5, …) is negative, while at even iterations (n = 2, 4, 6, etc.) $W_{TS}{}^n$ is positive. In effect, the resulting potential spatial decay curve sharpens, as the potential surrounding the peak gets significantly reduced (Right panel).

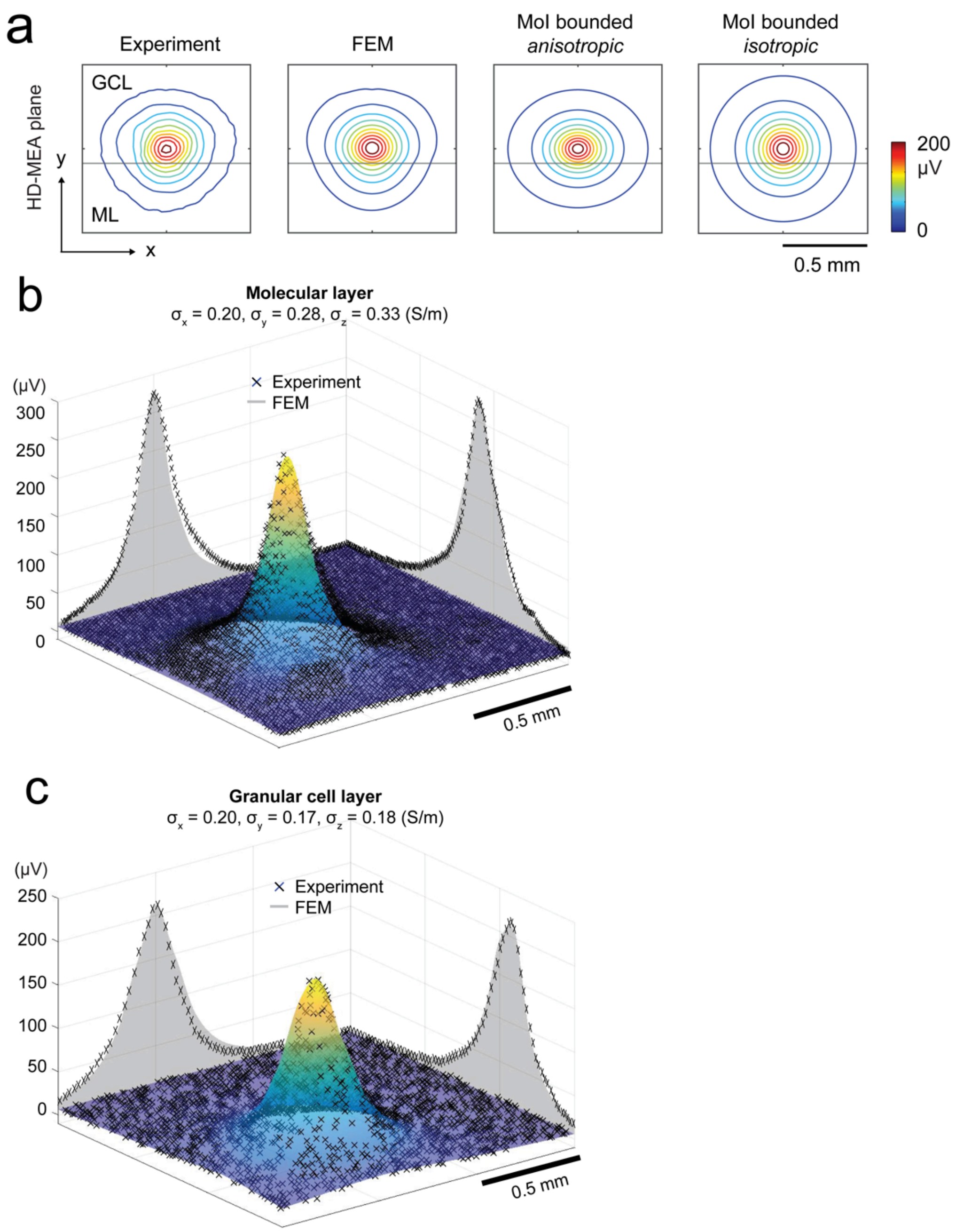

Supplementary Figure S06. Simulating the anisotropy of cerebellar brain slices. (a) 2D planar view of the potential spatial distribution of a point source located at the GCL or granular cell layer. The potential distributions of the experiment, FEM, MoI-bounded anisotropic and MoI-bounded isotropic simulation are shown. The 3D view of the potential spatial distributions comparing the FEM and experimental data are shown for a point source in (b) in the molecular layer (ML) and (c) in the granular cell layer (GCL).

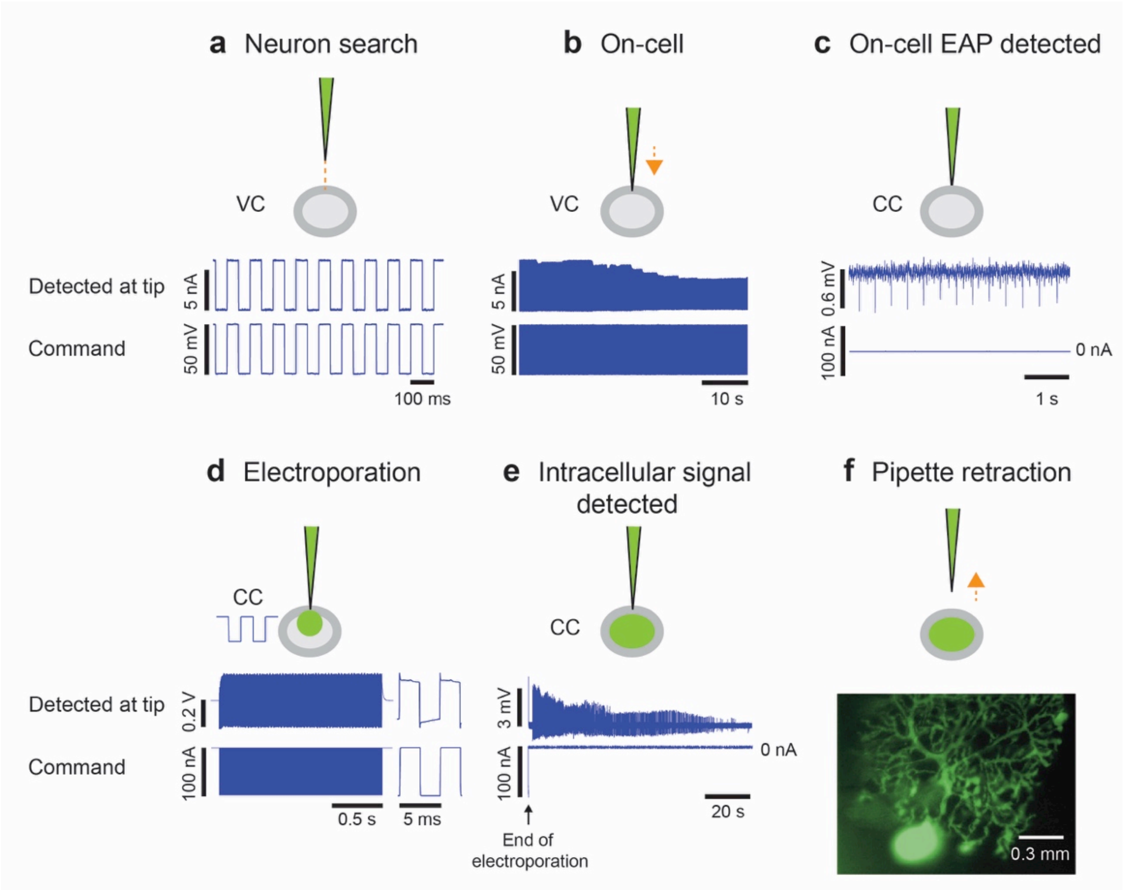

Supplementary Figure S07. Electroporation method applied to stain a neuron. The following steps were performed after placing the pipette at the approximate x-y location of the soma along the Purkinje cell layer. (**a**) The pipette was navigated deep into the tissue to search for a neuron. Positive pressure was applied to the pipette via a syringe. Using voltage-clamp (VC) mode, the pipette delivered a 50 mVpp square-wave signal at 1 kHz (command) and the current was detected at the tip of the pipette. (**b**) As the pipette approached the cell membrane, the detected current at the tip decreases, indicating an increase in pipette impedance. The pipette continued to move down towards the cell until the pipette impedance increased by 20-30%, which indicates that the pipette is on-cell. (**c**) Upon achieving on-cell state, the positive pressure applied to the pipette was released and the patch amplifier was set to current clamp (CC) mode. If the neuron was spontaneously active, extracellular action potentials (EAPs) could be detected at the pipette tip. (**d**) The cell was electroporated by stimulating the pipette with -100 nA current pulses at 200 Hz for 0.5 to 2 s. (**e**) After the stimulation, intracellular signals detected at the tip signified a successful electroporation. As the cell membrane recovered, intracellular signals decreased in amplitude until only extracellular signals could be detected. (**f**) Following the recovery of the cell membrane and waiting for a few minutes (2-3 minutes), the pipette was carefully retracted from the cell. The staining quality of the cell, inspected by using fluorescence microscopy, confirmed the success of the electroporation.
